# Computational Modeling of Electroencephalography and Functional Magnetic Resonance Imaging Paradigms Indicates a Consistent Loss of Pyramidal Cell Synaptic Gain in Schizophrenia

**DOI:** 10.1016/j.biopsych.2021.07.024

**Published:** 2022-01-15

**Authors:** Rick A. Adams, Dimitris Pinotsis, Konstantinos Tsirlis, Leonhardt Unruh, Aashna Mahajan, Ana Montero Horas, Laura Convertino, Ann Summerfelt, Hemalatha Sampath, Xiaoming Michael Du, Peter Kochunov, Jie Lisa Ji, Grega Repovs, John D. Murray, Karl J. Friston, L. Elliot Hong, Alan Anticevic

**Affiliations:** a Centre for Medical Image Computing and Artificial Intelligence, University College London, London, United Kingdom; b Institute of Cognitive Neuroscience, University College London, London, United Kingdom; c Wellcome Centre for Human Neuroimaging, University College London, London, United Kingdom; d Centre for Mathematical Neuroscience and Psychology and Department of Psychology, City University of London, London, United Kingdom; e Max Planck-UCL Centre for Computational Psychiatry and Ageing Research, London, United Kingdom; f Department of Psychiatry, Yale University School of Medicine, New Haven, Connecticut; g Picower Institute for Learning & Memory and Department of Brain and Cognitive Sciences, Massachusetts Institute of Technology, Cambridge, Massachusetts; h Department of Psychiatry, Maryland Psychiatric Research Center, University of Maryland School of Medicine, Baltimore, Maryland; i Department of Psychology, University of Ljubljana, Ljubljana, Slovenia

**Keywords:** Auditory steady-state, Dynamic causal model, Mismatch negativity, Psychosis, Resting state, Schizophrenia

## Abstract

**Background:**

Diminished synaptic gain—the sensitivity of postsynaptic responses to neural inputs—may be a fundamental synaptic pathology in schizophrenia. Evidence for this is indirect, however. Furthermore, it is unclear whether pyramidal cells or interneurons (or both) are affected, or how these deficits relate to symptoms.

**Methods:**

People with schizophrenia diagnoses (PScz) (*n* = 108), their relatives (*n* = 57), and control subjects (*n* = 107) underwent 3 electroencephalography (EEG) paradigms—resting, mismatch negativity, and 40-Hz auditory steady-state response—and resting functional magnetic resonance imaging. Dynamic causal modeling was used to quantify synaptic connectivity in cortical microcircuits.

**Results:**

Classic group differences in EEG features between PScz and control subjects were replicated, including increased theta and other spectral changes (resting EEG), reduced mismatch negativity, and reduced 40-Hz power. Across all 4 paradigms, characteristic PScz data features were all best explained by models with greater self-inhibition (decreased synaptic gain) in pyramidal cells. Furthermore, disinhibition in auditory areas predicted abnormal auditory perception (and positive symptoms) in PScz in 3 paradigms.

**Conclusions:**

First, characteristic EEG changes in PScz in 3 classic paradigms are all attributable to the same underlying parameter change: greater self-inhibition in pyramidal cells. Second, psychotic symptoms in PScz relate to disinhibition in neural circuits. These findings are more commensurate with the hypothesis that in PScz, a primary loss of synaptic gain on pyramidal cells is then compensated by interneuron downregulation (rather than the converse). They further suggest that psychotic symptoms relate to this secondary downregulation.

SEE COMMENTARY ON PAGE 167

Reduced excitatory synaptic gain (i.e., decreased slope of the presynaptic input–postsynaptic response relationship) is believed to be a primary deficit in schizophrenia (1,2). This reduction may primarily affect pyramidal cells (1) or inhibitory interneurons (3). For example, loss of cortical interneuron markers (in postmortem studies of people with schizophrenia diagnoses [PScz]) was originally thought to indicate a primary interneuron pathology, but recent work suggests that these markers are activity dependent, so their loss may reflect weaker pyramidal inputs (4). Decreased interneuron function in the disorder may thus be primary or a compensatory response to try to rebalance excitatory and inhibitory transmission in cortical circuits (5). These hypotheses are difficult to test in vivo, however.

Various mechanisms may reduce synaptic gain in schizophrenia: the most important is probably hypofunction of NMDA receptors (NMDARs) and their postsynaptic signaling cascade (1,2). Evidence for this comes from psychiatric genetics (6), magnetic resonance spectroscopy imaging (7), neuropathological studies (4), and animal models (8), but of these, only magnetic resonance spectroscopy is performed in humans in vivo, and its glutamatergic measures are difficult to interpret. Other neuromodulatory dysfunctions in schizophrenia [e.g., reduced cortical dopamine (9) or muscarinic receptors (10)] can be assessed more directly using positron emission tomography, but magnetic resonance spectroscopy and positron emission tomography are very indirect measures of synaptic gain.

An alternative way to investigate synaptic gain is by using electroencephalography (EEG) paradigms such as the mismatch negativity (MMN), an auditory oddball paradigm (11), and auditory steady-state response at 40 Hz (40-Hz ASSR); a paradigm inducing neural oscillations using a click train (12); or in the “resting state,” measuring with EEG (rsEEG) or functional magnetic resonance imaging (rsfMRI). PScz show robust reductions in 40-Hz ASSR (12) (d ≈ 0.6) and MMN (11) (d ≈ 1) responses, which may relate to diminished synaptic gain and decreased gain modulation (13), respectively, but these paradigms are not direct indices of synaptic gain.

Neural mass models of noninvasive data can be parameterized in terms of synaptic gain and these parameters estimated, for example, using dynamic causal modeling (DCM) (14), furnishing model-based biomarkers (15,16). This has several advantages: it can estimate subject-specific parameters and can fit evoked (e.g., MMN) and induced (e.g., 40-Hz ASSR or resting) EEG responses and rsfMRI, and thus explain responses to different paradigms in terms of common synaptic parameters, such as gain or self-inhibition on pyramidal cells or interneurons. Although fMRI models cannot incorporate detailed microcircuit parameters, due to fMRI’s coarse temporal resolution, they can assess local changes in excitability. Third, one can employ hierarchical modeling, e.g., using group-level parameters recursively to inform single-subject fits, for example, using parametric empirical Bayes (PEB) (17).

To date, DCM studies of PScz have used modest sample sizes and single paradigms but have found reasonably consistent results, e.g., cortical disinhibition in EEG (13,18, 19, 20) and rsfMRI (21) and diminished contextual gain modulation (13,19,22). Nevertheless, foundational questions remain, including the following: are well-replicated group differences between PScz and control subjects (Con) across paradigms all ascribable to the same model parameter(s)? How do symptoms in PScz relate to these parameters? Here, we address these questions using DCM across multiple EEG and fMRI paradigms, in PScz, Con, and first-degree relatives (Rel).

## Methods and Materials

**Figure 1:**
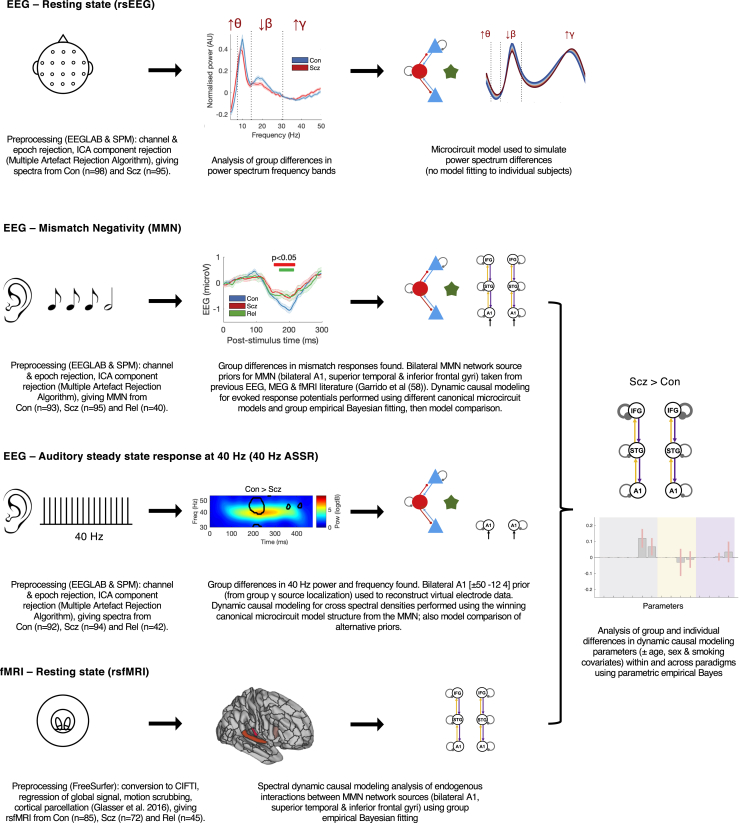
An overview of the analysis. This schematic illustrates the key steps in the preprocessing of the electroencephalography (EEG) (resting state [rs], mismatch negativity [MMN], and 40-Hz auditory steady-state response [ASSR]) and resting-state functional magnetic resonance imaging (rsfMRI) paradigms and their subsequent analysis using dynamic causal modeling (DCM) and parametric empirical Bayes. Simplified depictions of the paradigms are shown in the first column (see the Supplement for details), with group differences in EEG data features in the second column (first 3 rows) and DCM in the third column. The EEG data control group (Con) versus people with schizophrenia diagnoses (PScz) group differences are (from first to third rows) in rsEEG θ, β, and γ frequency band power (Figure 2A), MMN responses (Figure 3A), and 40-Hz ASSR power (Figure 4C). The second column of the final row (rsfMRI) shows the Glasser parcellation areas primary auditory cortex (A1) (middle), A4 (left), and 44 (right) containing the MMN sources A1, superior temporal gyrus (STG), and inferior frontal gyrus (IFG), respectively; these were used as nodes in the rsfMRI analysis, so that results could be compared across data modalities. Key preprocessing and analysis steps are described below the illustrations. DCM for EEG uses a cortical microcircuit model, shown on the left in the third column (also see Figure 2C). It contains superficial and deep pyramidal cells (blue triangles), inhibitory interneurons (red circles), and spiny stellate cells (green stars). The lower three DCM illustrations include macroscopic model structures, i.e., the cortical areas involved: A1, STG, and IFG (58). In the rsEEG analysis (top row), a single-area DCM was used to reproduce power spectra characteristic of each group. In the remaining paradigms, models were fitted to the data and parametric empirical Bayes was used to analyze group and individual differences. The final column depicts an example analysis (from Figure 3F) of group differences in DCM parameters between Con and PScz in the MMN. ICA, independent component analysis; MEG, magnetoencephalography; Rel, first-degree relative.

Data were collected from PScz (*n* = 107) recruited from outpatient clinics, first-degree relatives (*n* = 57), and control subjects (*n* = 108) recruited from media advertisements, who each underwent rsEEG, MMN, 40-Hz ASSR, and rsfMRI paradigms and recorded symptoms and other measures. PScz and Con were well matched in terms of age (mean ± SD = 39.4 ± 14.3 years and 39.4 ± 13.9 years, respectively), sex (59% and 68% male, respectively) and smoking status (33% and 39% smokers, respectively). PScz had mean Brief Psychiatric Rating Scale scores of 14.4 out of 49 for positive symptoms and 7.3 out of 28 for negative symptoms (Table S1). We first performed conventional analyses of group differences in data features for each paradigm. We then inferred the best explanations for these differences in terms of DCM parameters. Figure 1 summarizes the analysis (excluding results).

We used the DCM canonical microcircuit neural mass model (Figure S1) to analyze the EEG paradigms; more details are given in the Results, with a full description in the Supplement. Model parameters include connectivity strengths between populations, self-inhibition (synaptic gain) in these populations, and membrane time constants and transmission delays. For the rsEEG, MMN, and 40-Hz ASSR paradigms, we analyzed group differences using conventional data features (event-related potentials or power spectra). We then modeled either group-averaged data (rsEEG) or estimated subject-specific DCM parameters (MMN and 40-Hz ASSR). For rsfMRI, we only modeled the network generating MMN (and 40-Hz ASSR, in part) for comparative purposes.

We used PEB to analyze group and individual differences in synaptic (model) parameters, with the exception of rsEEG, where characteristic group responses were modeled. We interpret greater self-inhibition of pyramidal cells as an effective loss of pyramidal synaptic gain. Given known pathophysiology in PScz, NMDAR hypofunction seems the most likely explanation for loss of pyramidal gain, but other explanations are possible (see Supplement for further discussion).

Age, sex, smoking, and chlorpromazine dose equivalent covariates did not significantly affect the results, unless otherwise stated. All *t* tests were two-tailed, and rank sum tests were used if distributions were skewed; none are Bonferroni-corrected unless stated.

## Results

### In rsEEG, PScz Have Altered Power in θ, β, and γ Frequency Bands

**Figure 2:**
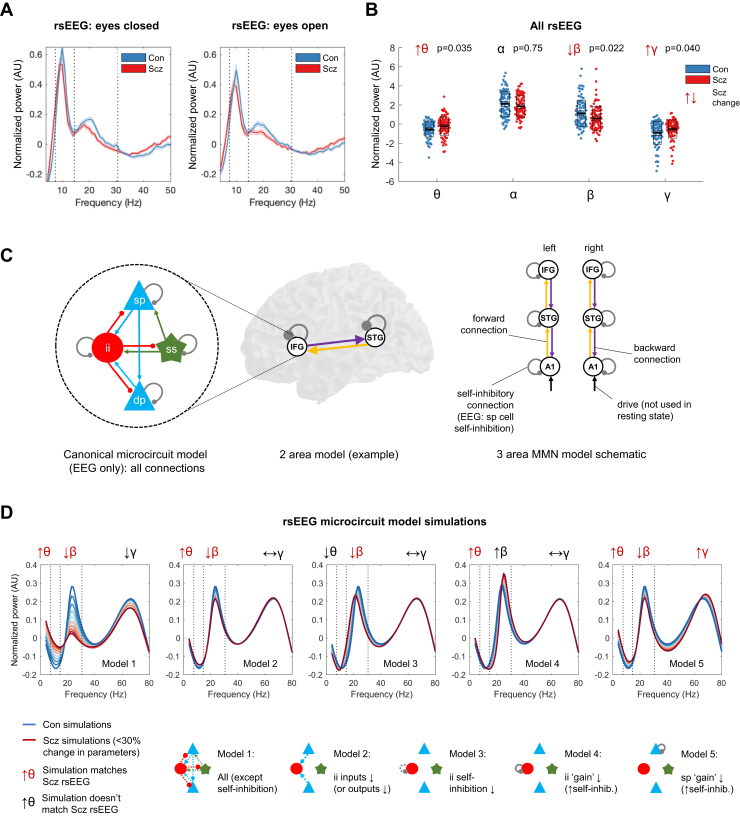
Resting-state electroencephalography (rsEEG) results, dynamic causal modeling (DCM) model structure, and rsEEG simulations. **(A)** Mean normalized eyes closed and eyes open rsEEG power spectra (± SEM) across all channels for control subjects (Con) (*n* = 98; blue) and people with schizophrenia diagnoses (PScz) (*n* = 95; red) groups, divided into 4 frequency bands (dotted lines): θ (3–7 Hz), α (8–14 Hz), β (15–30 Hz), and γ (>31 Hz). **(B)** Group comparisons in mean power across both eyes closed and eyes open conditions in the θ, α, β, and γ bands are shown. The box plots show the mean, SEM, and SD. *p* values are Bonferroni-corrected for 4 comparisons. **(C)** EEG DCMs used the current version of the canonical microcircuit model (59) (also see Figure S1A). This microcircuit (left) consists of superficial pyramidal (sp) and deep pyramidal (dp) cells, inhibitory interneuron (ii), and spiny stellate (ss) cells. They are interconnected with excitatory (arrowheads) and inhibitory (beads) connections; their self-inhibitory connections parameterize their responsiveness to their inputs, i.e., synaptic gain. In EEG DCM, each modeled cortical area contains a microcircuit (middle); functional magnetic resonance imaging DCM uses a much simpler neuronal model. Both DCMs have self-inhibition parameters (round gray beads), which—in EEG—inhibit superficial pyramidal cells specifically. A schematic DCM diagram is explained on the right. **(D)** The top row shows the results of 5 sets (models 1–5) of simulations of microcircuit parameter changes and their similarity to the rsEEG changes in θ, β, and γ bands in PScz (the model does not produce an α peak). The parameters changed in each model are illustrated in the microcircuit schematics for models 1–5 (bottom row); parameter increases are denoted by whole lines and decreases by dotted lines. Each model is used to produce 10 simulations, starting with standard parameter values (to simulate Con) plotted in dark blue, and then reducing or increasing the parameters illustrated below in increments of 3% to simulate PScz (up to the most extreme change, plotted in dark red). Only model 5, an increase in superficial pyramidal self-inhibition, i.e., a loss of synaptic gain, reproduces the changes seen in all 3 frequency bands. IFG, inferior frontal gyrus; MMN, mismatch negativity; STG, superior temporal gyrus.

We first examined rsEEG power spectra by subtracting the 1/f gradient, noting that gradients did not differ between groups with eyes open or closed (*p* > .2). The mean adjusted power spectra within the Con (*n* = 98) and PScz (*n* = 95) groups are shown in Figure 2A, for eyes closed (left) and open (right) conditions, with θ/α/β/γ frequency bands demarcated. A repeated-measures analysis of variance (between-subjects factor, group; within-subjects factors, eyes open/closed and frequency band) demonstrated a significant interaction of frequency × group (*F*_3,573_ = 6.59, *p* < .001) but not of eyes × group (*F*_1,191_ = 0.05, *p* = .8) or of frequency × eyes × group (*F*_3,573_ = 0.4, *p* = .8). We therefore averaged the power in each frequency band across eyes open and closed conditions and performed Wilcoxon rank sum tests (as some distributions were skewed), Bonferroni-corrected for 4 frequency bands (Figure 2B). PScz had increased θ (*Z* = 2.63, *p*_Corr_ = .035), decreased β (*Z* = −2.77, *p*_Corr_ = .022), and increased γ (*Z* = 2.58, *p*_Corr_ = .040), but unchanged α (*Z* = −1.32, *p*_Corr_ = .75).

### Increased Pyramidal Self-inhibition Explains θ, β, and γ Changes in PScz

We used DCM’s canonical microcircuit model—a biophysical model of interacting pyramidal, interneuron, and spiny stellate populations (Figure 2C, left)—to identify the most likely synaptic pathology. To model power spectrum changes in PScz, we treated cortex as a single microcircuit in which specific parameters were changed in 5 plausible ways (Figure 2D, bottom): a loss of all microcircuit connectivity (model 1), a loss of pyramidal connections to or from interneurons (model 2), interneuron disinhibition (model 3), increased interneuron self-inhibition (model 4), and increased pyramidal cell self-inhibition (model 5). Note that this model does not fit the large α peak.

Only model 5 could explain the θ, β, and γ changes seen in PScz (Figure 2D, upper row). Models 1 and 2 only reproduced the θ and β changes. Model 3 showed decreased β peak frequency, which was quantitatively lower in PScz but not statistically significant (Figure S2A).

### MMN and P100 Are Reduced in Both PScz and Rel

**Figure 3:**
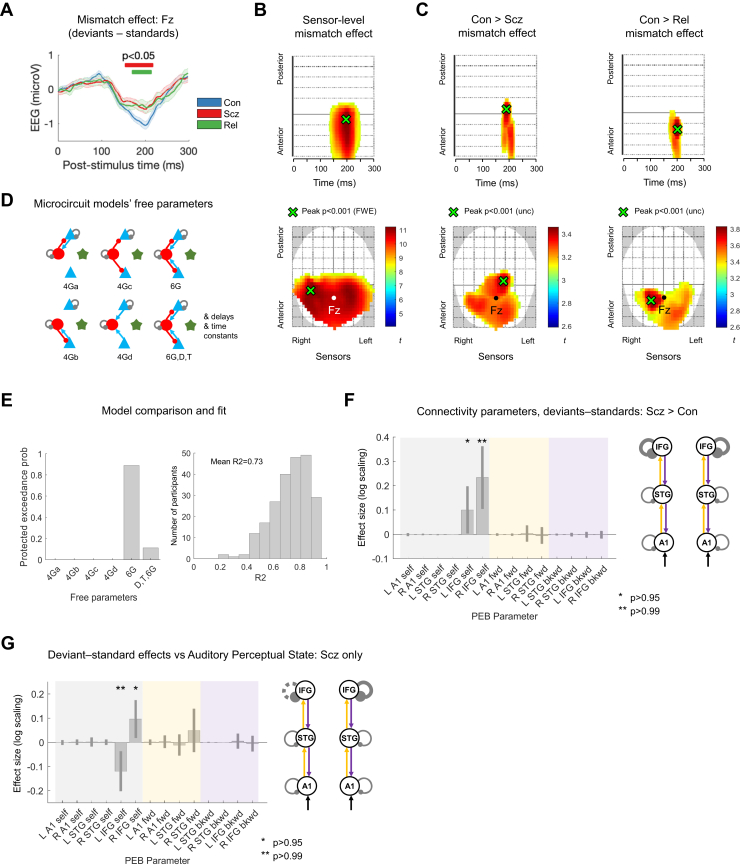
Mismatch negativity data and modeling analysis. **(A)** Mismatch difference waves (i.e., deviant–standard, mean ± SEM) for control subjects (Con) (*n* = 94; blue), people with schizophrenia diagnoses (PScz) (*n* = 96; red), and first-degree relatives (Rel) (*n* = 42; green) at electrode Fz. Group differences are computed using *t* tests (uncorrected [unc]) at each time point and are marked with red (PScz vs. Con) and green (Rel vs. Con) bars above the difference waves. There were no significant PScz vs. Rel differences. **(B)** The lower plot shows the location of the mismatch effect (i.e., deviants—standard) at sensor level across all Con and PScz, displayed at *p* < .05 (familywise error [FWE]). Fz is shown in white. The peak effect is shown in green (*p* < .001 [FWE], *t*_376_ = 11.23). The upper plot shows sensors vs. time; the peak effect occurs at 198 ms. **(C)** These plots show the interaction of condition and group for the Con > PScz contrast (left) and Con > Rel contrast (right) in the same format as Figure 2B, at the lower threshold of *p* < .005 (unc) for display purposes. Both groups demonstrate similar differences from Con in the mismatch contrast in frontocentral sensors just before 200 ms. **(D)** Microcircuit models were compared, differing only in which parameters were allowed to change from their priors (estimated G connectivity parameters are shown, as in Figure 2C). These models’ free G parameters included various combinations of superficial pyramidal and/or deep pyramidal cell (blue) connections to or from inhibitory interneurons (red) and self-inhibition of superficial pyramidal and inhibitory interneuron cells. Note that each parameter within each microcircuit could differ between subjects but was constrained to be the same in every cortical area within subjects, except for superficial pyramidal self-inhibition, which could differ throughout. The final model also estimated delay (D) and time constant (T) parameters (these were fixed in the other five models). **(E)** Model comparison and evaluation. Left: the protected exceedance probability is the probability a particular model is more likely than any other tested model, above and beyond chance, given the group data. The model with most free parameters is at the far right; it comes second to the 6G model with fixed delays and time constants and 6 microcircuit connectivity parameters estimated. Right: a histogram of *R*^2^ values for all participants for the winning model; it fits most participants well. **(F)** A parametric empirical Bayes (PEB) analysis of mismatch negativity model parameters (i.e., connections) that contribute to the PScz > Con mismatch effect. The results are plotted on the left (with 95% Bayesian confidence intervals) and shown in schematic form on the right; parameters with posterior probabilities of *p* > .95 or *p* > .99 of contributing to the group difference effect are indicated with 1 (∗) or 2 asterisks (∗∗), respectively. On the plot, self-inhibitory connections are shaded gray, forward (fwd) connections shaded yellow, and backward (bkwd) connections shaded purple (matching the colors in the schematic). The y-axis denotes log-scaling of the effect size; changes of exp (±0.2) are of roughly ±20%. Some parameters have been eliminated during Bayesian model reduction (see Supplement). The analysis indicates that PScz showed greater self-inhibition (or reduction in synaptic gain) in bilateral inferior frontal gyrus (IFG) in the mismatch contrast. The Rel > Con contrast did not show significant effects. **(G)** PEB analysis of mismatch negativity mismatch effect model parameters that correlate with current (state) abnormal auditory percepts within PScz only, plotted in the same format as Figure 3F. Within PScz, abnormal auditory percepts relate to reduced self-inhibition in right (R) IFG but disinhibition in left (L) IFG (in Broca area). All effects shown in **(F)** and **(G)** are also present without the addition of age, sex, and smoking covariates (*p* > .95). Inclusion of a chlorpromazine dose equivalent covariate renders the analysis in **(F)** nonsignificant (*p* > .75), but it makes the overall effect of PScz on L and R IFG self-inhibition become significant (Figure S4C). STG, superior temporal gyrus.

The MMN paradigm consisted of standard and duration-deviant tones. The mismatch amplitude is the deviant–standard response in electrode Fz (11), which was reduced in both PScz and Rel around 200 ms (Figure 3A). There were no significant group differences in MMN latency between Con (mean ± SD latency = 194 ± 34 ms) and Rel (196 ± 45 ms, *p* = .8) or PScz (202 ± 44 ms, *p* = .18). In the averaged deviant and standard waveforms (Figure S2B), PScz showed reduced response amplitudes around 50 to 100 ms in both, and an exaggerated mismatch-like response around 175 ms in the standard condition.

Smoothed sensor-level data were analyzed using cluster-based statistics. Across Con and PScz, there was a strong mismatch effect, peaking at 198 ms (peak familywise error-corrected *p* [*p*_FWE_] < .001, *t*_376_ = 11.23) (Figure 3B), which was reduced in PScz (peak at 186 ms, *p*_unc_ < .001, cluster *p*_FWE_ = .010, *t*_376_ = 3.46) and in Rel (peak at 198 ms, *p*_unc_ < .001, cluster *p*_FWE_ = .011, *t*_268_ = 3.83) (Figure 3C). Likewise, PScz had a reduced P100 response (peak at 82 ms, *p*_FWE_ = .003, cluster *p*_FWE_ < .001, *t*_376_ = 4.83), as did Rel, although this was only significant at an uncorrected peak threshold (peak at 94 ms, *p*_unc_ = .001, cluster *p*_FWE_ = .8, *t*_268_ = 3.02) (Figure S2C).

### DCM of MMN Indicates Increased Frontal Self-inhibition in PScz, but Disinhibition in Broca Area Relates to Abnormal Auditory Percepts

We first used model comparison to establish whether it was best to fix or estimate various microcircuit parameters in the MMN analysis (see Supplement). We compared 6 models (Figure 3D): model 6G estimates 6 connectivity (G) parameters, models 4Ga-d consider subsets of these six, and model 6G,D,T also estimates delays and time constants. Bayesian model selection preferred model 6G (also in Con and PScz separately), with a protected exceedance probability of *p* = .89 (Figure 3E, left). This model fitted most participants’ data accurately (e.g., Figure S3A). A histogram of *R*^2^ values is shown in Figure 3E (right); the group mean *R*^2^ was 0.73. *R*^2^ were slightly higher in Con (mean ± SD = 0.76 ± 0.13) than in PScz (0.70 ± 0.14; rank sum *Z* = 3.12, *p* = .0018) and Rel (0.71 ± 0.15; rank sum *Z* = 2.14, *p* = .033) (Figure S3C).

We then used PEB to ask which parameters best explained group differences in MMN: self-inhibition within areas or connections between areas. The reduced mismatch amplitude in PScz was best explained by increased self-inhibition in deviant—relative to standard—trials in left (L) inferior frontal gyrus (IFG) (*p* > .95) and right (R) IFG (*p* > .99) (Figure 3F). Including chlorpromazine dose equivalent covariates reduced the posterior probability to *p* > .75, but age, sex, and smoking had no effect. Conversely, there was no overall group effect (across both standards and deviants) of PScz on the microcircuit parameters (all *p* < .95) (Figure S4C, left) unless chlorpromazine dose equivalents were included as covariates; here, PScz showed greater superficial pyramidal self-inhibition in L and R IFG (both *p* > .99) (Figure S4C, middle and right) and reduced interneuron self-inhibition throughout (*p* > .95). Rel did not show effects of *p* > .95 in either analysis.

In PScz, the auditory perceptual abnormalities state measure was associated with disinhibition in L IFG (*p* > .99)—within the Broca area—but increased self-inhibition in R IFG (*p* > .95) in the mismatch contrast (Figure 3G). Historical auditory perceptual abnormalities (the trait measure) showed similar effects but at lower posterior probability (*p* > .75, not shown).

### PScz Had Reduced γ Power and Peak Frequency in 40-Hz ASSR, and Rel Had Reduced γ Power

**Figure 4:**
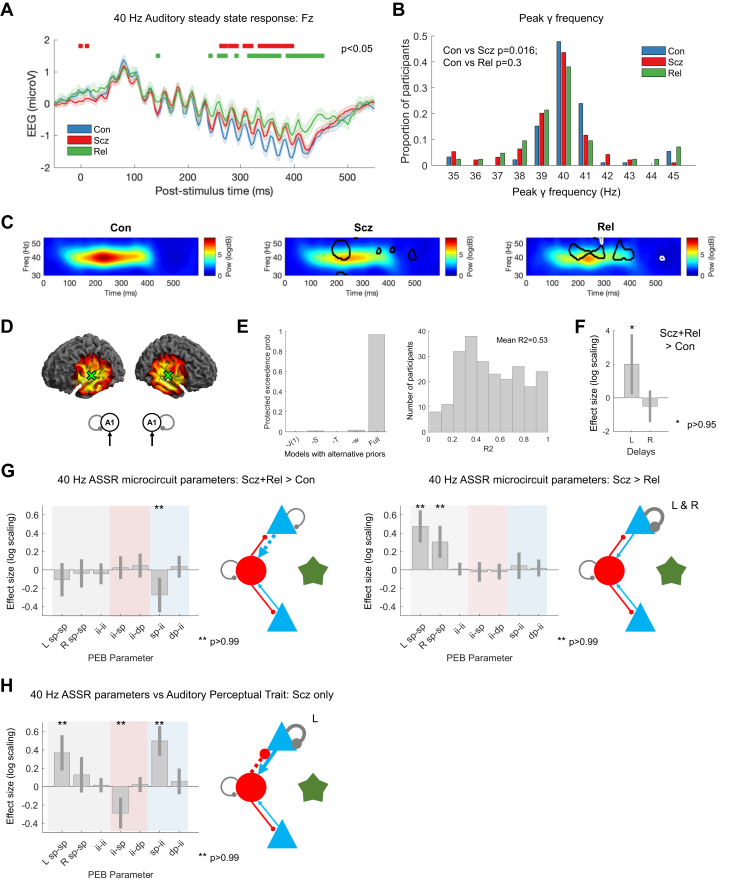
40-Hz auditory steady-state response (ASSR) data and modeling analysis. **(A)** 40-Hz ASSR time courses at electrode Fz for control subjects (Con) (*n* = 92; blue), people with schizophrenia diagnoses (PScz) (*n* = 94; red), and first-degree relatives (Rel) (*n* = 42; green). Sixteen clicks were played at 40 Hz, starting at 0 ms. Group differences in the baseline deflection (not modeled subsequently) emerge after around 250 ms, shown with red bars (Con vs. PScz) and green bars (Con vs. Rel), both *p* < .05 (*t* tests per time point, uncorrected). **(B)** γ (35–45 Hz) frequencies with the strongest power (in the normalized spectrum) in each participant are shown in a histogram. **(C)** These normalized time frequency plots show the ∼40 Hz responses around 100 to 400 ms. The PScz and Rel plots have areas of difference from Con encircled in black; the Rel plot has areas of difference from PScz encircled in white (*p* < .05, *t* tests at each time and frequency). **(D)** The left plots show the bilateral primary auditory cortex (A1) (transverse temporal gyrus) sources chosen following source localization [±50 -12 4]. The 40-Hz ASSR model structure is on the right: bilateral sources in A1. **(E)** Left: to improve the dynamic causal modeling fit of the cross spectral densities in bilateral A1 in this nonstandard paradigm, we used empirical priors (also see Figure S1A) for J(1), the contribution spiny stellate cells make to the electroencephalography (EEG) signal; S, the gain of the neuronal activation function; T, population time constants; and w, the width of the ∼40 Hz Gaussian bump. The plot shows that the full model (with all the empirical priors) is superior to other models that used standard values for their respective priors (or for -w, 1 Hz instead of 4 Hz). Right: a histogram of *R*^*2*^s for all participants for the winning model. **(F)** Parametric empirical Bayes (PEB) analysis indicated that PScz + Rel > Con showed increased neural transmission delays in left (L) A1. **(G)** Left: PEB analysis (in the same format as Figure 3H) indicated that PScz + Rel > Con (a psychosis genetic risk effect) had decreased superficial pyramidal (sp)–inhibitory interneuron (ii) connectivity. Right: PScz > Rel (a psychosis diagnosis effect) shows decreased sp self-inhibition in bilateral A1. **(H)** PEB analysis in PScz, showing that abnormal auditory percepts are associated with disinhibition of the sp-ii circuit (and increased sp self-inhibition in L A1). All effects shown in **(F)**, **(G)**, and **(H)** are also present without the addition of age, sex, and smoking covariates (*p* > .95) and with inclusion of chlorpromazine dose equivalents as a covariate. dp, deep pyramidal; Freq, frequency; Pow, power; R, right.

We next considered induced responses during auditory steady-state stimulation. Group-averaged 40-Hz ASSR are shown in Figure 4A and the distributions of participants’ peak γ (35–45 Hz) frequencies in Figure 4B. PScz had slightly reduced γ peak frequency: mean peak frequencies (following subtraction of the 1/f gradient) (Figure S2E) were Con = 40.2 Hz (SD, 1.7), PScz = 39.5 Hz (SD, 1.7; *t*_184_ = 2.67, *p*_Corr_ = .016), and Rel = 39.9 Hz (SD, 2.1; *t*_132_ = 1.03, *p* = .3). Adjusted time-frequency plots are shown in Figure 4C (and raw time-frequency data in Figure S2F): Con showed a robust increase in ∼40 Hz power around 100 ms, which is diminished in PScz and Rel (*p* < .05; *t* tests at each frequency and time point are circled on the middle and right plots, for Con vs. PScz and Con vs. Rel in black and PScz vs. Rel in white; this many differences are unlikely due to chance—Con vs. PScz and Con vs. Rel both *p* < .001, PScz vs. Rel *p* = .006, permutation tests). Maximum ASSR γ power correlated with MMN amplitude in PScz (*r* = 0.28, *p*_Corr_ = .029) but not in Con (*r* = 0.04, *p* = .7) or Rel (*r* = 0.14, *p* = .4).

### 40-Hz ASSR DCM Suggests a Loss of Pyramidal Input to Interneurons in PScz and Rel and Greater Self-inhibition in PScz

The peak cortical source—closest to primary auditory cortex (A1)—was (50 −12 4), hence bilateral sources at (±50 −12 4) were used as priors for reconstruction of virtual electrode data: the DCM comprised these bilateral sources and their thalamic drive (Figure 4D). Empirical priors for several parameters were used to optimize model fit (Figure S1A). Bayesian model comparison between the full model (containing empirical priors for the contribution of spiny stellate cells to measured signals, the neural activation function, and synaptic time constants) and models with standard priors for these parameters showed that the full model was superior (Figure 4E, left). The 40-Hz thalamic drive was modeled using a Gaussian bump function of width *w* ≤ 4 Hz (see Supplement); this width performed better than a narrower bump of 1 Hz (model -w) (Figure 4E). Model fits for the winning model were reasonable (mean *R*^2^ = 0.53) (Figure S3B). Group differences in *R*^2^ were not detected (rank sum tests: all *p* > .1) (Figure S3C).

We performed group comparisons with PEB using schizophrenia genetic risk (PScz + Rel > Con) and diagnosis (PScz > Rel) as explanatory variables (13,19), instead of PScz > Con and Rel > Con comparisons (as in the MMN analysis). This was because the group differences in data features were less marked in the 40-Hz ASSR, and there were substantial differences between Rel and Con parameters, only some of which were shared by PScz (Figure S6B). The genetic risk effect was an increased conduction delay in L A1 (*p* > .95) (Figure 4F), and reduced superficial pyramidal (sp) to inhibitory interneuron (ii) connectivity (*p* > .99) (Figure 4G, left). The schizophrenia diagnosis effect was increased superficial pyramidal self-inhibition in bilateral A1 in PScz (both *p* > .99) (Figure 4G, right).

### 40-Hz ASSR DCM Links Abnormal Auditory Percepts to A1 Disinhibition in PScz

In PScz, the auditory perceptual abnormalities trait measure related to a disinhibited sp-ii-sp circuit, i.e., increased sp-ii (*p* > .99) and reduced ii-sp connectivity (*p* > .99), and greater self-inhibition in L A1 (*p* > .99) (Figure 4H). The auditory state measure had similar associations but at lower posterior probability (*p* > .95 for sp-ii, *p* > .75 for ii-sp and sp-sp: not shown).

### rsfMRI DCM of the MMN Circuit Finds Increased Self-inhibition in IFG in PScz and Rel

We then analyzed effective connectivity within the MMN network during rsfMRI, i.e., the Glasser parcellation areas (in the rsfMRI data) based on MMN source locations (see Supplement): bilateral A1, A4, and 44 (Figure 1). The microcircuit model for fMRI data is simpler than the neural mass models used for EEG; however, they retain inhibitory self-connections. Model fits were accurate: *R*^2^s were >0.7 in all groups, with no group differences (rank sum tests: all *p* > .05) (Figure S3C).

**Figure 5:**
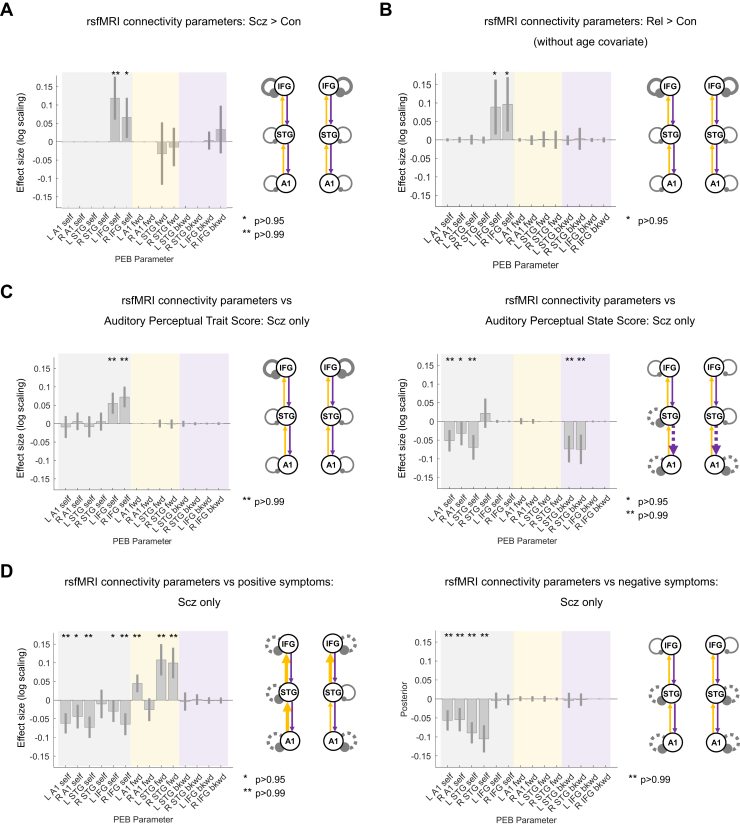
Resting-state functional magnetic resonance imaging (rsfMRI) modeling analysis. **(A)** For comparative purposes, the rsfMRI connectivity analysis was conducted on the same network as the mismatch negativity (MMN) analysis. Results for control subjects (Con) (*n* = 85) and people with schizophrenia diagnoses (PScz) (*n* = 72) are shown in the same format as Figure 3F. As in the MMN, PScz showed increased self-inhibition in the bilateral inferior frontal gyrus (IFG). Inclusion of chlorpromazine equivalent dose as a covariate still showed increased self-inhibition in left (L) IFG but only at *p* > .75. **(B)** rsfMRI connectivity analysis without covariates for Con (*n* = 85) and first-degree relatives (Rel) (*n* = 45) is shown. Similar to PScz, Rel show increased self-inhibition in the bilateral IFG, but this effect disappeared with addition of the age covariate (*p* < .75). **(C)** Left: within PScz, abnormal auditory percepts (trait measure) related to increased self-inhibition in the bilateral IFG. Right: conversely, abnormal auditory percepts (state score, i.e., experiences within the last week only) relate to disinhibition in temporal areas and also a loss of top-down connections within the auditory cortex. The right (R) primary auditory cortex (A1) effect was attenuated if age, sex, and smoking covariates were not included and if a chlorpromazine dose equivalent covariate was added. **(D)** Left: within PScz, Brief Psychiatric Rating Scale positive symptom score related to disinhibition throughout the MMN network and increased forward (fwd) connectivity in 3 of 4 connections. Most effects were robust to addition of chlorpromazine dose equivalents as a covariate (all *p* > .99 except L IFG self-inhibition, *p* > .75), removal of the hallucinations score from the Brief Psychiatric Rating Scale positive symptom total (all *p* > .95 except L IFG and R A1 self-inhibition, *p* > .75), and analysis without covariates (all *p* > .99 except L IFG self-inhibition, *p* > .75). Right: within PScz, Brief Psychiatric Rating Scale negative symptom score related to disinhibition in temporal nodes of the MMN network. All effects shown (except Rel > Con) are also present without the addition of age, sex, and smoking covariates and if participants (2 Con, 8 PScz) with rsfMRI signal-to-noise ratio <25 are excluded (all *p* > .95). Some rsfMRI results are no longer significant without global signal regression (Figure S7). No results change substantially with inclusion of chlorpromazine dose equivalent as a covariate unless stated. bkwd, backward; PEB, parametric empirical Bayes; STG, superior temporal gyrus.

In PEB analysis, PScz showed increased self-inhibition in L and R IFG (*p* > .99 and *p* > .95, respectively) (Figure 5A). These effects were robust to age, sex, and smoking covariates (and to the removal of the 10 participants with the lowest rsfMRI signal-to-noise ratio: 8 PScz and 2 Con; both *p* > .95). These effects did not survive addition of chlorpromazine dose equivalents (L IFG self-inhibition fell to *p* > .75). However, Rel > Con showed the same increase in self-inhibition in bilateral IFG (both *p* > .95) (Figure 5B). This group difference did not survive addition of the age covariate: Rel were older than Con (Rel mean age = 45.4 ± 16.6 years, Con mean age = 39.4 ± 14.3 years; *t*_162_ = 2.4, *p* = .02). These differences were not detected using conventional functional connectivity analyses, which cannot assess self-inhibition, or analyses of regional variance (Figure S6B–E and Supplement for further discussion).

### rsfMRI DCM Reveals Relationships of Positive Symptoms to Cortical Disinhibition in PScz

PEB analysis within PScz found that trait auditory perceptual abnormalities were associated with increased self-inhibition in L and R IFG (both *p* > .99) (Figure 5C, left). Conversely, state auditory perceptual abnormalities were associated with disinhibition in R A1 (*p* > .95) and L A1 and superior temporal gyrus (STG) (both *p* > .99) and STG-A1 backward connectivity bilaterally (both *p* > .99) (Figure 5C, right).

Similarly, Brief Psychiatric Rating Scale positive symptoms (including age, sex, smoking, and negative symptoms covariates) were associated with decreased self-inhibition everywhere except R STG (all *p* > .99 except L IFG and R A1, both *p* > .95) and stronger forward connections everywhere except R Al-STG (all *p* > .99) (Figure 5D, left). Brief Psychiatric Rating Scale negative symptoms (including age, sex, smoking, and positive symptom covariates) were associated with decreased self-inhibition in all temporal—but not frontal—nodes (all *p* > .99) (Figure 5D, right).

Note that many rsfMRI results were lost if global signal regression was not performed (Supplemental Results, Figure S7).

### Self-inhibition Findings in PScz Across Electroencephalography and rsfMRI Paradigms Are Similar

**Figure 6:**
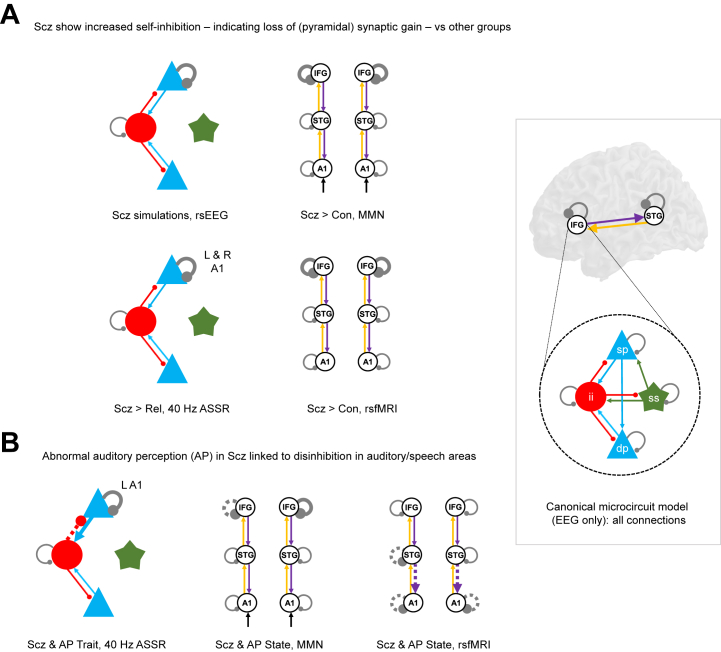
Summary of key findings across paradigms. This figure illustrates similar dynamic causal modeling findings across paradigms using the schematic illustrations from previous analyses. The inset at bottom right shows the canonical microcircuit model for electroencephalography (EEG) (below), which exists in each modeled cortical area (above). The microcircuit consists of superficial pyramidal (sp) and deep pyramidal (dp) cells (blue), inhibitory interneuron (ii) (red), and spiny stellate (ss) cells (green), interconnected with excitatory (arrowheads) and inhibitory (beads) connections. **(A)** Crucially, the people with schizophrenia diagnoses (Scz) group consistently exhibited increased self-inhibition (as expected from a loss of synaptic gain) in superficial pyramidal cells in particular (i.e., in the EEG paradigms). This was the case (from left to right) in primary auditory cortex (A1) in the 40-Hz auditory steady-state response (ASSR) (when compared with first-degree relatives [Rel]), in the bilateral inferior frontal gyrus (IFG) in both the mismatch negativity (MMN) (deviant–standard contrast) and the resting-state functional magnetic resonance imaging (rsfMRI), and in the rsEEG simulations. **(B)** Within the PScz group, abnormal auditory percepts were linked with disinhibition in A1 in both the 40-Hz ASSR paradigm and the rsfMRI and with disinhibition in left (L) IFG—i.e., Broca area—in the MMN (deviant–standard contrast). Con, control subjects; R, right; STG, superior temporal gyrus.

In summary, we found clear evidence for increased self-inhibition (evidence of reduced synaptic gain) in PScz (Figure 6A) in all data modalities and paradigms. However, auditory perceptual abnormalities within PScz were associated with the opposite change: disinhibition (Figure 6B). A sensitivity analysis (see Supplement) confirmed that increased superficial pyramidal self-inhibition best reproduced the key data features of MMN (i.e., decreased MMN amplitude but unchanged latency) (Figure S8A) and, along with loss of sp-ii connectivity, decreased 40-Hz ASSR (Figure S8B). Evidence for within-subject correlations in self-inhibition parameters across paradigms was weak, however (see Supplemental Results, Figure S9).

## Discussion

DCM of EEG and fMRI produced two key cross-paradigm findings. First, well-established effects in rsEEG (23), MMN (11), and 40-Hz ASSR (12) paradigms in PScz were replicated, and all could be explained by increased self-inhibition in (superficial) pyramidal cells. Likewise, PScz also showed an increase in prefrontal self-inhibition—similar to MMN—in rsfMRI (Figure 6A). This strongly favors the hypothesis that there is diminished synaptic gain on pyramidal cells (1,2,5) over the hypothesis of diminished synaptic gain on interneurons (3) in this sample of PScz with established illness.

Second, abnormal auditory percepts in PScz were associated with decreased self-inhibition in auditory areas selectively across 3 paradigms (Figure 6B). This is consistent with 40-Hz ASSR γ power (24) [and phase locking of auditory γ (25)] correlating positively with auditory symptoms, despite being reduced in PScz overall [as in the visual domain (26)], and with hallucinations and psychotic-like experiences relating to decreased self-inhibition in IFG across the psychosis spectrum (27). Positive symptoms were also associated with disinhibition in the rsfMRI analysis (Figure 5D). These opposing effects of group and symptoms on self-inhibition (28)—and also on cortical glutamate (29)—support the hypothesis (1,5) that decreased synaptic gain (NMDAR hypofunction in particular) is compensated by allostatic disinhibition of pyramidal cells (i.e., interneuron downregulation) and, furthermore, indicate that psychotic symptoms result from this disinhibitory rebalancing of excitatory and inhibitory transmission.

In rsEEG, increased θ power in PScz is a well-established finding (23,30). A U-shaped change in spectral power (here, increased θ, decreased β, increased γ) has been seen several times across θ, α, and β frequencies (23). Increases (not decreases) in α and β in PScz have been seen in eyes open rsEEG (30,31), but in unnormalized data; before subtracting the 1/f gradient, β power was numerically higher in our sample of PScz as well. This speaks to the importance of distinguishing band-specific changes from changes in 1/f slope, which itself is increased by lower excitation:inhibition ratio (32,33). Of note, low γ (30–45 Hz) power is typically reduced in PScz with longstanding diagnoses (34), but we lacked illness duration information.

Decreased mismatch amplitude in PScz [and especially in chronic PScz (35)] is well documented (11), and we found an effect of similar size in Rel, larger than is typical (35). Underlying this effect, we found that deviant stimuli decrease self-inhibition in IFG in Con but not in PScz, recapitulating other DCM studies (13,22). The mismatch amplitude rarely correlates with hallucinations in PScz [e.g., in only 3 of 22 studies (11)], but we found abnormal auditory percepts related to (condition-specific) disinhibition in L IFG—Broca’s area. Traditional MMN analysis (using electrode Fz) might miss this lateralized effect. Nevertheless, there are reports of left-lateralized (including IFG) associations of hallucinations with auditory oddball responses in PScz (36).

In the 40-Hz ASSR, PScz showed decreased γ power and peak frequency, and Rel showed decreased power [as elsewhere (12,20,37)]. DCM indicated that diminished pyramidal connectivity to interneurons (and greater transmission delay) was common to both PScz and Rel, but loss of pyramidal gain was unique to PScz (Figure 4G). Others have modeled 40-Hz ASSR in PScz by increasing interneuron time constants (38); this reproduced a concurrent increase in 20-Hz power in PScz (38), which was not observed in our data. We assumed time constants did not differ in PScz in the ASSR or MMN, and estimated connectivity parameters—and delays, in the ASSR—instead (these can be regarded as synaptic rate constants).

A previous rsfMRI DCM analysis in PScz found disinhibition in the anterior cingulate cortex (21), rather than increased self-inhibition in bilateral IFG (Figure 5A). This recalls a pattern of altered intraprefrontal functional connectivity in early PScz (39): increased connectivity of medial areas and more modest decreases in connectivity in lateral areas. Prefrontal hyperconnectivity correlated positively with positive symptoms (39). We similarly found that positive symptoms were associated with disinhibition in bilateral IFG and A1 (Figure 5D, left). This relationship echoes findings that increased functional connectivity of primary sensory areas (to the thalamus) correlates with Positive and Negative Syndrome Scale scores (40), and that increased A1 rsfMRI autocorrelation (a result of reduced self-inhibition) in PScz relates to auditory hallucinations (28) (Figure 5C, right). Our results have commonalities with a spectroscopy mega-analysis that correlated positive symptoms to frontal and negative symptoms to temporal glutamate concentrations (29) (Figure 5D). Thus, symptoms may depend not just on connectivity between nodes but on synaptic gain within nodes; modeling is key to disambiguating these possibilities.

More data are required to draw firm conclusions about the Rel group. In MMN, no effects exceeded *p* > .95 despite Rel’s similar data features to PScz. In 40-Hz ASSR, pyramidal self-inhibition was reduced in Rel (Figure S5B), not increased. In rsfMRI, however, Rel showed comparable IFG self-inhibition increases to PScz (Figure 5B).

A crucial question is what changes in self-inhibition mean: changes in synaptic gain or reciprocal coupling with interneurons? Our interpretation of self-inhibition changes is guided by known pathophysiology in PScz, i.e., given that cortical synaptic gain is decreased [e.g., reduced function of NMDA (1,2,6), dopamine D_1_ (9), and muscarinic (10) receptors] and inhibitory interneurons downregulated (4,5), then the most logical interpretation of increases and decreases in pyramidal self-inhibition is diminished pyramidal synaptic gain (41,42) and decreased interneuron function, respectively (gain in the neural mass model is discussed in detail in the Supplement.) If the fundamental pathology in PScz was a loss of synaptic gain on interneurons, one would expect to see consistent group effects of increased interneuron self-inhibition and/or decreased pyramidal self-inhibition, neither of which were found.

Regarding potential causes of reduced synaptic gain, some PScz data features imply NMDAR hypofunction. In rsEEG, increased γ follows NMDAR antagonism (43), e.g., using ketamine (which also suppresses β) (44), or in NMDAR encephalitis (which also increases θ) (15,45). In contrast, LSD and psilocybin do not increase θ (46), and D_2_ antagonists potentiate α and β (47,48). The 40-Hz ASSR is sensitive to NMDAR function (49) but also cholinergic (50), dopaminergic (51) and serotonergic (52) manipulations; the latter do not affect MMN, however, which is quite specific to NMDAR function (11). Ketamine also reduces rsfMRI functional connectivity of IFG and auditory cortices (53). Antipsychotic dose covariates weakened the PScz MMN condition-specific effects (Figure 3F) but strengthened the overall MMN effects (Figure S4C); they also weakened the PScz rsfMRI effects, but similar rsfMRI effects emerged in unmedicated Rel (Figure 5). Overall, these findings resemble NMDAR hypofunction and seem unlikely to be medication induced.

Several limitations are addressable. Given that pathophysiology is dynamic in PScz (1) and that subgroups may exist (54), larger datasets should be analyzed, containing more early-course (and preferably unmedicated) PScz. Notably, even the latter show reductions (*d* > 1) in cortical glutamate (55), consistent with the idea that pyramidal cell hypofunction—rather than disinhibition—is primary in PScz. DCM models with explicitly parameterized NMDA (and other) receptor conductances (15) can explore self-inhibition in more detail and across more cortical areas.

In conclusion, we found consistently increased self-inhibition (i.e., diminished synaptic gain) in PScz, especially in frontal areas, but disinhibition—in auditory areas in particular—correlated with auditory perceptual abnormalities. Psychotic symptoms may therefore be caused by interneuronal downregulation that restores cortical excitation/inhibition balance in PScz. These complex processes may explain why successful glutamatergic treatments for PScz are elusive, and why such treatments may have narrow therapeutic windows (56) or depend on illness stage (57).

## Supplementary Material

Supplementary Material

Key Resources Table
