# Supplementary material for: Computational Modeling of Electroencephalography and Functional Magnetic Resonance Imaging Paradigms Indicates a Consistent Loss of Pyramidal Cell Synaptic Gain in Schizophrenia
Source: Biol Psychiatry. 2022 Jan 15;91(2):202–15. doi: 10.1016/j.biopsych.2021.07.024 (PMC8654393; doi:10.1016/j.biopsych.2021.07.024)
Supplement: Supplementary Material [file mmc1.pdf]

Computational Modeling of Electroencephalography and Functional Magnetic Resonance Imaging Paradigms Indicates a Consistent Loss of Pyramidal Cell Synaptic Gain in Schizophrenia

Supplement

Supplementary Methods

Supplementary Results

Supplementary Figures S1-S10

Table S1

Supplementary Methods

Participant characteristics and measures

Data were collected from a single site (Maryland Psychiatric Research Center) from studies sponsored by the National Institute of Health (MH085646 and DA027680) and under human subject protocols approved by the University of Maryland, Baltimore, Institutional Review Board (HP-00045716 and HP-00043082). Analysis was approved by University College London Research Ethics Committee (4356/003). All participants gave written informed consent prior to participation. Data were collected between December 2010 and April 2016. The study participants comprised those with a diagnosis of schizophrenia (PScz, n=107), controls who were approximately matched for age, gender and smoking status (n=108), and first degree relatives of PScz (n=57): details of the groups are listed in Table S1. Diagnoses were made using the Structured Clinical Interview for DSM Diagnoses (SCID) (1). PScz were recruited from outpatient clinics, relatives from PScz participants themselves and also from media advertisements, and controls from media advertisements. Exclusion criteria were: neurological illness, head injury, substance abuse or dependence (except for nicotine), and daily use of benzodiazepine drugs. Controls and relatives were excluded if they met criteria for an Axis I diagnosis using the SCID, and controls were required to have no family history of psychosis within two generations.

All participants completed the Digit Symbol Substitution Task (2) – a symbol-copying task that assesses learning, executive function and processing speed – and the Auditory Perceptual Trait and State scale (APTS) (3). The APTS is a self-rated scale that assesses the frequency of abnormal auditory percepts, from altered characteristics of sounds to illusions, pseudohallucinations and verbal and non-verbal hallucinations. The scale includes ‘trait’ and ‘state’ measures, defined as symptoms experienced over one’s lifetime until two weeks ago, and over the past week, respectively. The full scale is available at http://www.mdbrain.org/APTS.pdf. Its test–retest reliability was assessed in 41 participants about 4 months apart, which showed ICC = 0.81 for both the trait and state measures, suggesting good reliability. (Much more questionnaire and imaging data were collected, e.g. about visual perceptual abnormalities, anxiety, depression, nicotine dependence, working memory (digit sequencing), and further EEG auditory steady state and working memory

paradigms, but these data were not analysed in this study. We focused on the measures of auditory perceptions and processing speed as these have the largest effect sizes in PScz (4)).

Use of psychotropic medication was recorded. 90 PScz were taking one or more antipsychotics (including Clozapine; see Table S1), for 15 PScz no medication was recorded and 3 were unmedicated. Some participants were also taking antidepressants, hypnotics (as prescribed) or mood stabilisers – the PScz group had higher proportions in each case. The 20 item Brief Psychiatric Rating Scale (BPRS) was used to rate overall symptoms in the PScz group only.

Data acquisition and paradigms

For EEG recording, participants sat in a semi-reclining chair inside a sound-attenuated chamber, wearing an electrode cap. 64 scalp electrode sites were recorded, according to the 10/20 International System, using Neuroscan Stim acquisition software and a Synamps2 or Synamps2 RT amplifier. Recordings were grounded midway between FPZ and FZ using silver/silver-chloride electrodes and referenced to the nose. Eye movements were monitored by vertical and horizontal electrooculograms (EOGs). Data were continuously sampled at 1000 Hz with a DC/100 Hz band-pass filter (24 dB/octave roll-off). Impedances were kept below 5 kΩ.

For the resting state EEG (rsEEG) paradigm, subjects were asked to remain awake whilst EEG data were recorded. Two recordings were obtained on the same day, one with eyes open, and one with eyes closed. Each recording lasted 5 minutes.

In the mismatch negativity (MMN) paradigm, participants were presented with 1000 auditory stimuli through earphones, of which 800 (80%) were standard tones presented at 75 dB, for 60 msec, at 1000 Hz; 200 (20%) were duration-deviant tones at 75 dB, for 150 msec, at 1000 Hz. All tones had a 5 msec rise/fall time, with a stimulus onset asynchrony of 300 msec. 800 tones were presented in a single block. Participants were asked to ignore the tones while viewing a silent movie. Some of the MMN data have been analysed elsewhere (5), using classical statistical tests and structural equation modeling to find correlations between MMN

responses and neurotransmitter levels. Here we obtained effective connectivity estimates using DCM.

In the auditory steady state response (ASSR) paradigm, participants listened to click trains delivered by headphones, at 2.5, 5, 10, 20, 40, and 80 Hz. 75 stimulus trains (trials) – each consisting of 15 clicks, with each click at 72 dB and of 1 ms duration – were delivered at each stimulus frequency. The duration ranged from 6 s per train for 2.5 Hz, to 0.1875 s per train for 80 Hz. The inter-train interval was 0.7 s. Therefore, the durations for 2.5, 5, 10, 20, 40, and 80 Hz were 8.38, 4.69, 2.82, 1.89, 1.42, and 1.19 min, respectively, presented in 6 separate blocks separated by 2 min. The order of the blocks was randomized. In this study, only the data for the 40 Hz ASSR were analysed: data from this and the other frequency bands have been analysed elsewhere (3).

For the resting state fMRI (rsfMRI) paradigm, participants were asked to keep their eyes closed and relax. fMRI data were collected at the University of Maryland Center for Brain Imaging Research using a Siemens 3T TRIO MRI (Erlangen, Germany) system equipped with a 32-channel phase array head coil. Resting state functional Blood Oxygen Level Dependent (BOLD) T2*-weighted images were obtained with axial slices parallel to the anterior-posterior commissure (AC-PC) using a T2*-weighted gradient-echo, echo-planar sequence (TR=2.2 s, TE=2.8 ms, flip angle=13°, FOV=256 mm, 128×128 matrix, 1.94x1.94 in-plane resolution, 4mm slice thickness, 37 axial slices, 444 volumes per run; 1 run). Acquisition lasted around 16 minutes. Structural images were acquired using a T1-weighted, 3D magnetization-prepared rapid gradient-echo (MPRAGE) sequence (TR/TE/TI=2200/4.13/766 ms, flip angle=13°, voxel size [isotropic]=0.8 mm, image size=240x320x208 voxels), with axial slices parallel to the AC-PC line.

Data preprocessing

All EEG preprocessing and analysis was performed within Matlab (The MathWorks Inc.). MMN and 40 Hz ASSR data were imported into EEGLAB (6) (https://sccn.ucsd.edu/eeglab/) and band-pass filtered between 1 and 70 Hz, with a notch filter at 59.5-60.5 Hz. rsEEG data were band-pass filtered between 1 and 50 Hz as higher γ frequencies are more vulnerable to artefacts in non-timelocked data. The data were then epoched: rsEEG into 6 s epochs, MMN

into epochs from -50 ms to 300 ms around the stimulus onset, and 40 Hz ASSR data into epochs from -100 ms to 600 ms around the stimulus onset. The data then underwent an automated artefact rejection pipeline within EEGLAB, also employing the Multiple Artefact Rejection Algorithm (MARA) (7). First, epochs with amplitudes of >±5 std were rejected, as were epochs containing linear trends (using pop_rejtrend, max slope=5, min R2=0.7) and low (0-2 Hz) or high (20-40 Hz) frequency bands (using pop_rejspec) within power thresholds of -50 to 50 dB and -100 to 25 dB respectively (40 Hz ASSR data used a frequency band of 20-35 Hz given the click train was presented at 40 Hz). Channels were then rejected (using pop_rejchan) on the basis of extreme values in their spectrum (-4 to 6 std), kurtosis (-7 to 15 std) or joint probability (-9 to 7 std). If >50% epochs were rejected, channel rejection was performed first, and epoch rejection second. If >50% epochs were still rejected, or if >20% channels were rejected, the data was discarded. For the MMN, 17/246 subjects ultimately had >50% epochs rejected but no subjects had >20% channels rejected. For the 40 Hz ASSR, 12/259 subjects ultimately had >50% epochs rejected but no subjects had >20% channels rejected.

Independent component analysis (ICA) was then performed, and components with p>0.5 of being artefacts – determined using stored artefact templates within MARA – were then rejected. MARA contains a supervised machine learning algorithm that learns from expert ratings of 1290 components by extracting features from the spectral, spatial and temporal domains, in order to identify artefacts of all kinds (e.g. eye, muscle, loose electrodes, etc). After MARA, channel and epoch rejection were run a second time using lower thresholds, as sometimes artefacts became apparent following component rejection. Channels were rejected on the basis of the following values in their spectrum (-6 to 5 std), kurtosis (-6 to 9 std) or joint probability (-7 to 7 std). Missing channels were then interpolated and the data were rereferenced to the common average. The data were then converted to SPM format and imported into SPM12 (v7219) (8), available at https://www.fil.ion.ucl.ac.uk/spm/software/spm12/. The MMN and 40 Hz ASSR data were baseline corrected to the first 50 ms of the epoch, and the MMN data were downsampled to 250 Hz. The MMN (standard and deviant conditions) and 40 Hz ASSR were then averaged within each subject using robust averaging (using default options for spm_robust_average, which preserves roughly 95% of data points).

The structural MRI and rsfMRI data were preprocessed in accordance with the Human Connectome Project (HCP) Minimal Preprocessing Pipelines (MPP), adapted to be compatible with “legacy” data (single-band BOLD, no field map or T2-weighted image). These modifications to the MPP are described in detail in prior publications (9) and are offered as a standard HCP MPP option at https://github.com/Washington-University/HCPpipelines/pull/156. Briefly, the T1-weighted images were first aligned in the volume space to the standard Montreal Neurological Institute-152 (MNI-152) brain template via a single-step combined transformation using the FMRIB Software Library (FSL) linear image registration tool (FLIRT) and non-linear image registration tool (FNIRT) (10). FreeSurfer (http://surfer.nmr.mgh.harvard.edu/) was then used to segment gray and white matter in the whole brain to produce individual cortical-subcortical anatomical segmentations (11) – specifically the pial and white matter cortical boundaries which were used to define a “cortical ribbon”, and a subcortical grey matter voxel mask. These were then used to define the hybrid surface/volume neural file for each subject in the Connectivity Informatics Technology Initiative (CIFTI) “grayordinate” space (12). All images for all subjects were visually inspected to assess the T1-weighted image quality and FreeSurfer grey/white matter segmentation performance. The T1-weighted images were checked for ringing, blurring due to motion, signal inhomogeneity and other artefacts or anomalies. If severe enough to impair segmentation, the subject was omitted. Otherwise, if segmentation failed in a cortical area in >2 consecutive slices, the segmentation was repeated with adjusted parameters and reassessed. If segmentation failure persisted, the subject was omitted. This rigorous procedure excluded 67/269 subjects (Table S1).

BOLD images were first slice time corrected using FSL slicetimer. Next, motion correction was performed by aligning all BOLD data to the first frame of every run via McFLIRT. Motion corrected BOLD images were then registered to T1w image using FreeSurfer BBR. Finally, all the linear transformation matrices and nonlinear warp images were combined and the original slice time corrected BOLD images were registered to MNI-152 brain template in a single step. To exclude signal from non-brain tissue a brainmask was applied and cortical BOLD data were converted to the CIFTI gray matter matrix by sampling from the anatomically-defined cortical ribbon. These cortical data were then aligned to the HCP atlas using surface-based nonlinear deformation (12). Subcortical voxels in the BOLD data were extracted using the Freesurfer-defined segmentation to isolate the subcortical volume portion of the CIFTI space.

In addition to the HCP MPP, all BOLD images had to pass stringent quality assurance criteria as previously reported (13) to ensure that all functional data were of comparable and high quality: i) signal-to-noise ratios (SNR) >90, computed by obtaining the mean signal and standard deviation (sd) for a given slice across the BOLD run, while excluding all non-brain voxels across all frames (14); ii) movement scrubbing as recommended by Power et al (15,16). 'Movement scrubbing' refers to the practice of removing BOLD volumes that have been flagged for high motion, in order to minimize movement artefacts, and is a widely used fMRI preprocessing technique. Specifically, to further remove head motion artefacts, as accomplished previously (13), all image frames with possible movement-induced artefactual fluctuations in intensity were identified via two criteria: First, frames in which sum of the displacement across all 6 rigid body movement correction parameters exceeded 0.5 mm (assuming 50 mm cortical sphere radius) were identified; second, root mean square (RMS) of differences in intensity between the current and preceding frame was computed across all voxels divided by mean intensity and normalized to time series median. Frames in which normalized RMS exceeded 1.6 times the median across scans were identified. The frames flagged by either criterion were marked for exclusion (logical or), as well as the one preceding and two frames following the flagged frame. Movement scrubbing was performed for all reported analyses across all subjects. Subjects with more than 50% of frames flagged were removed from subsequent analyses. Next, to remove spurious signal in resting state data we completed additional preprocessing steps, as is standard practice (17): all BOLD time-series underwent high pass temporal filtering (>0.008 Hz), removal of nuisance signal extracted from anatomically-defined ventricles, white matter, and the remaining brain voxels (i.e. global signal) (all identified via participant-specific FreeSurfer segmentations (18)), as well as 6 rigid-body motion correction parameters, and their first derivatives using previously validated in-house Matlab tools (19). Note that while the removal of global signal is a debated topic in fMRI (20,21), it remains the gold standard for removing spatially pervasive artefact in the brain (although other techniques are emerging (22,23)). BOLD signal within the subject-specific cortical mask was spatially smoothed with a 6 mm full-width-at-half-maximum (FWHM) Gaussian kernel and dilated by two voxels (6 mm) to account for individual differences in anatomy, as done previously (14,24). The cortical data were then parcellated using the 360-area multi-modal parcellation from Glasser et al. (2016) (25).

Data analysis and modelling: resting state EEG

The power spectrum for each electrode in each subject was computed using the default settings of Welch's method in Matlab (6 s windows with overlaps of 3 s). Each power spectrum was normalised (sensor by sensor) by subtracting the 1/f slope in log space: this gradient was determined using the default settings (bisquare weight function) of robustfit in Matlab. The normalised spectra were averaged over all sensors and all subjects in each group. Group differences in normalised power in each frequency band were computed using the following definitions: θ 3-7 Hz, α 8-14 Hz, β 16-30 Hz, γ 31-50 Hz.

The biophysical model used to simulate power spectra is a convolution-based neural mass model (26) in DCM. It is a neural mass model in the sense that it treats ensemble neural activity as a point process (without spatial extension) and only first order statistics (means) of neural population activities influence each other. Convolution-based models (as opposed to conductance-based models, which model ion channels in cell membranes) convolve presynaptic inputs (firing rates) with synaptic response kernels to produce postsynaptic membrane potentials in a given neural population (27,28). The parameters summarising the kernel are the maximum postsynaptic depolarisation (determined by receptor density, or 'connectivity') and membrane time constants. These parameters are listed as connectivity parameters G and time constants T in Figure S1A, and the equations of the model given in Figure S1B. The model transforms the neural population's membrane potential back into a firing rate using a sigmoid operator whose slope is controlled by parameter S. This firing rate becomes the input for another population, determined by the network's intrinsic and extrinsic connectivity. Delays in transmission within the microcircuit (intrinsic) and from one brain area to another (extrinsic) are parameterised by D (not shown). The microcircuit itself (Figure S1A) contains four neural populations: spiny stellate cells (ss), superficial pyramidal cells (sp), deep pyramidal cells (dp) and inhibitory interneurons (ii). Extrinsic connections follow known anatomical patterns (29): 'forward' connections project from sp cells to ss and dp cells in the area above, and 'backward' connections project from dp cells to sp and ii cells in the area below. Given all of the above, the dynamic activity of all populations in the network can be computed for any given input (for evoked responses like the MMN) or as a filtered spectral response to endogenous neuronal fluctuations (a mixture of pink and white neuronal noise) as in the rsEEG (26).

There are several forms of 'gain' within a convolutional neural mass model (illustrated in Figure S1B). One is 'somatic gain', the slope S of the sigmoid function σ converting membrane potential to firing rate. Another is 'synaptic gain', determined by the height (due to connectivity, G) and spread of the synaptic kernel g. To assess the synaptic gain of sp and other cells in this work, we use the self-inhibitory G parameters (e.g. sp-sp) for that population, because these apply irrespective of the afferent inputs (unlike other G parameters, e.g. ss-sp, which pertain to specific connections).

Note, however, that self-inhibitory connections may parameterise one or more of several physiological effects: i) they control the responsiveness of a population to its inputs, as any mechanism controlling synaptic gain does, e.g. NMDAR function, but also classical neuromodulatory receptors such as cholinergic and dopaminergic receptors, or the connectivity between neurons within a pyramidal population; ii) they may reflect the action of an inhibitory interneuron population in a circuit, e.g. from sp cells projecting to parvalbumin+ (PV^+) interneurons and back to sp cells (30); iii) in the case of interneurons, they may reflect autapses (self-synapses: common on PV^+ cells (31)). A model cannot distinguish between these mechanisms unless they are explicitly encoded in the model itself; however, some interpretations are more or less likely given what we know of pathology in schizophrenia. For example, a finding of increased sp self-inhibition in PScz is more likely to be due to loss of synaptic gain in those cells than a strengthening of the sp-PV^+-sp circuit, which most evidence implies is weakened in PScz (32). The opposite is true for a finding of decreased sp self-inhibition in PScz: its most likely explanation would be a loss of ii inhibition of sp cells.

Similarly, the microcircuit model does not explicitly distinguish between PV^+ and somatostatin+ (SST^+) interneurons. SST^+ interneurons are of interest as their cortical markers are just as (if not more) reduced in PScz as PV^+ (33). That said, the dynamics of the sp self-inhibition connection in the model are faster than those of the sp-ii-sp circuit (see the parameters in Figure S1A), hence the model can potentially distinguish between faster (likely PV^+) and slower (e.g. SST^+) interactions between pyramidal cells and interneurons (30). (Note however that the empirical priors used for the 40 Hz ASSR analysis accelerated the dynamics of the sp-ii-sp circuit, in order to model the 40 Hz peak.)

The microcircuit model used was the same as that used by Shaw et al (34): we termed this spm_fx_cmc_2017. It makes a small adjustment to spm_fx_cmc_2014, in that it replaces a G connection from sp to ss with a connection from sp to dp: see Figure 2C and Figure S1A. We used this model because it is closer to known anatomy (29) than previous versions – e.g. having distinct but connected superficial and deep pyramidal populations that send ascending and descending projections respectively – but not too complicated to fit to EEG data (e.g. more sophisticated models (35) contain separate interneuron pools for sp and dp cells, and are conductance-based, i.e. contain parameters for specific receptors, but they are harder to fit to EEG (36)). Given our primary interest was in the function of (and connectivity between) pyramidal cells and interneurons in PScz, this model was most suitable (although conductance-based models may furnish more useful pharmacological biomarkers). One key aspect of the model is that it models cortical dynamics only, and hence can model the γ and β peaks generated by superficial and deep cortical layers (respectively) (37–39), but it cannot reproduce an α peak without adding an additional (thalamic) input (40,41). Given the absence of group differences in α in the rsEEG, and for simplicity, we did not model this frequency band (as in previous studies (34,36)).

To simulate the power spectra in controls and PScz, we used the SPM12 (v7219) function spm_induced_optimise, which computes transfer functions (i.e. representations of cortical dynamics in the spectral domain) for parameters in the biophysical models in DCM. The simulated spectra were normalised in exactly the same way as the empirical spectra, subtracting the 1/f gradient using robustfit in Matlab. The standard priors in DCM were used (Figure S1A).

We did not try to fit the microcircuit model to the empirical rsEEG data (although this is possible (36)) because the choice of sources in rsEEG data can be problematic. We instead used the microcircuit model to simulate the effects of various potential circuit pathologies in PScz, and compared the results to the pattern in the rsEEG. The circuit pathologies were based on reasonable hypotheses about microcircuit connectivity abnormalities in PScz (Figure 2D):

Model 1 – a <30% loss in connectivity in all microcircuit connections, i.e. a global loss of synaptic efficacy.

Model 2 – a <30% loss of connectivity to/from interneurons (‘to’ and ‘from’ are identical from the modelling point of view – their effects are the same).

Model 3 – a <30% loss of interneuron self-inhibition, to model potential disinhibition of PV+ interneurons by other PV+ interneurons (31).
Model 4 – a <30% gain in interneuron self-inhibition, to model loss of synaptic gain (e.g. hypofunction of NMDARs) on interneurons.
Model 5 – a <30% gain in superficial pyramidal self-inhibition, to model loss of synaptic gain (e.g. hypofunction of NMDARs) on sp cells.

Data analysis and modelling: mismatch negativity

The MMN data were plotted (using electrode Fz, as is standard practice (42)) as group-averaged waveforms for the standard and deviant tones separately (Figure S2B) and as the traditional 'difference' waves, i.e. deviant-standard waves, illustrating the mismatch effect (Figure 3A). Group differences in these waveforms were assessed using *t*-tests at each timepoint using an α of $p<0.05$ (uncorrected for multiple comparisons), and are displayed on the figures.

To more robustly illustrate the mismatch effects and group differences, incorporating other electrodes and cluster-based correction for multiple comparisons, the MMN sensor-level data were analysed in SPM12, after smoothing using an 8x8x8 mm FWHM Gaussian kernel (Figures 3B, 3C and S2C).

The sources of the MMN response are well characterised, e.g. using conjoint EEG-fMRI studies (43). These are bilateral sources in primary auditory cortex (A1), secondary auditory cortex (superior temporal gyrus, STG) and inferior frontal gyrus (IFG). To ensure these source locations were adequate for our modelling, we source localised the MMN data using the 'minimum norm' model in SPM12. Priors on source locations (in MNI coordinates) were [-42, -22, 7] – left A1, [46, -14, 8] – right A1, [-61, -32, 8] – left STG, [59, -25, 8] – right STG, [-46, 20, 8] – left IFG, [46, 20, 8] – right IFG, and sources were estimated to be within a 16 mm radius of these prior locations. The activity peaks across all subjects and conditions were close to the standard sources (Figure S2D). Note that this source reconstruction was not used for the DCM analysis, however: source reconstruction (using the same priors) was performed as part of DCM model inversion. Joint optimisation of source locations and model parameters performs better than separate optimisations because it suppresses posterior

correlations between source location parameters and biophysical parameters such that they explain away different parts of the data.

Standard DCM analyses estimate subject-specific parameters in four microcircuit connections (G): sp-sp, ii-ii, ii-sp and ii-dp. We estimated two extra G parameters given the strong possibility of differences in connectivity from pyramidal cells to interneurons in PScz (32), i.e. sp-ii and dp-ii. To avoid overparameterising the model, however, we fixed parameters elsewhere. First, we estimated whole group posterior means for synaptic delays (D) and time constants (T) and then used these as priors, fixing the D and T parameters to these values (Figure S1A) in most models. Insodoing, we are not claiming that D and T are unlikely to be altered in PScz. We are merely assuming that there are very likely to be differences in G parameters in PScz, and that we wish to give the model the best chance of detecting them by reducing its degrees of freedom elsewhere. Second, we constrained all estimated G parameters to be the same (within but not between subjects) in every cortical area except for sp-sp self-inhibition. There are two reasons to adopt this approach: i) it regularises the model by allowing all cortical areas to inform the same parameters, reducing the overfitting that would occur when estimating six times as many parameters from one sixth of the amount of data per parameter, ii) post mortem studies of interneurons in PScz (33,44) indicate that pathology is broadly distributed across the cortex rather than focused in a single area, as one might expect from a disorder involving many genes of small effect. Nevertheless, NMDAR (and other receptor) subtypes are differentially distributed along a cortical hierarchical gradient (45), so if for example certain subtypes – preferentially located in higher or lower hierarchical areas – are more affected in PScz (46), the model could detect these spatial differences in sp-sp connectivity. Furthermore, sp-sp connectivity could also differ between standard and deviant conditions, permitting the model to infer condition-specific neuromodulatory effects on pyramidal cells (e.g. from NMDA (47) or cholinergic (48) receptors). Given we were applying a brain-wide constraint to most G parameters, we used the whole group posterior means (Figure S1A) as priors, to reduce model fitting problems.

In total, six microcircuit models were evaluated (shown in Figure 3D):

Models 4Ga, 4Gb, 4Gc, 4Gd – these four models estimated four microcircuit connectivity (G) parameters each, consisting of sp-sp and ii-ii self-inhibitory connections and then different combinations of two of the four connections between sp, ii and dp cells. Delays (D) and time constants (T) were fixed to their group posterior estimates (see Figure S1A).

Model 6G – this model estimated all six G parameters of interest, but fixed D and T to their group posterior estimates.
Model 6G,D,T – this model estimated all six G parameters of interest and also D and T (using their group posterior estimates as priors).
Each of these models used the same macroscopic structure (Figure 2C, right), i.e. forward and backward connections linking areas in adjacent hierarchical levels, and lateral connections linking bilateral areas at the same level (not shown). Note that only forward, backward and self-inhibitory connections could show *condition-specific* differences between groups, i.e. differences between standard and oddball event related potentials.

MMN model fitting was performed using the 'spatial' or 'IMG' forward model in DCM for evoked response potentials (ERPs). DCM fits the first up to eight modes of the prior predicted covariance in sensor space (49) (Figure S3A). In practice, the first 3-4 modes usually capture most of the variance (as in Figure S3A, right), so R^2 values generated for the MMN data are based only on the first four modes. A boundary elements head model (50) was used to approximate the brain, cerebrospinal fluid, skull and scalp surfaces.

Fitting non-linear neural mass models to EEG data is an ill-posed problem, and can lead to difficulties in optimisation, such as models getting stuck in local optima. Empirical Bayes for DCM (51) can circumvent this issue by performing model fitting iteratively, each time using the group level posteriors over parameters as priors for each subject's parameters in the next model inversion, yielding more robust and efficient parameter estimates (52). In practice, it substantially improved model fit: model inversions performed with and without it had R^2 values of ~0.7 and ~0.6 respectively. Local optima can also be avoided by fitting one fully parameterised model and then pruning unnecessary parameters using Bayesian model reduction (see below), rather than fitting many models with reduced numbers of parameters which are more prone to local optima problems (53). One subject failed model inversion (added to 17 who failed preprocessing: Table S1).

Following model inversion, models were formally compared using Bayesian model selection (54) (Figure 3E). We used the protected exceedance probability (55) as the metric of success: i.e. the probability that any one model is more frequent than the others, above and beyond chance. The R^2 for the first four modes of the winning model was also computed (Figure 3E).

Group differences between Con and PScz or Rel in their R^2 values were also assessed using ranksum tests (as the distributions were skewed; Figure S3C).

Group differences in parameters and relationships between parameters and other measures were analysed using Parametric Empirical Bayes (PEB; spm_dcm_peb) (53,56). PEB is a hierarchical Bayesian version of a general linear model that can assess which DCM parameters differ between groups or covary with a continuous measure. One critical advantage of PEB (over performing classical statistical tests on parameters) is that it takes account of not just each parameter's expectation but also its covariance: therefore parameters that cannot be estimated with high confidence (e.g. in models that fit less well) contribute less to the inference. PEB is superior to standard model inversion at both selecting the correct model and estimating the model parameters (using simulated data) (51).

A second key aspect of the PEB procedure is the use of Bayesian model reduction (57) and Bayesian model averaging (53) (spm_dcm_peb_bmc). In essence, these steps prune away unnecessary parameters and then obtain posterior estimates for the remaining parameters by averaging over the remaining models[1]. The Bayesian model reduction procedure allows hypotheses about model parameters to be formally tested. This is done by first defining a model space of different parameter groupings that may best describe the modelled effect. In the MMN analysis, two model spaces were used: the first asked which combinations of *extrinsic or self-inhibitory* connections could best account for the *mismatch* effect (Figure S4A, left), and the second asked which combinations of *intrinsic* (microcircuit) connections could best account for the *overall group differences* across conditions (Figure S4B, left). These model spaces were based on the assumption that microcircuit parameters would not differ between conditions, but messages passed between areas (or pyramidal gain in specific areas) may well do so. In Figure S4A the first model (column) is a 'null' model, the second contains only 'forward' connections, the third contains only 'backward' connections, etc. The

[1]Note that a correction for multiple comparisons is only appropriate when using classical statistics to reject the null hypothesis. In Bayesian inference (including PEB) there is no intimation that any hypothesis is rejected – the inferences are based upon the relative evidence or marginal likelihood for the hypotheses in question. In other words, if an effect has a posterior probability of 95%, this means there is a 5% possibility that the effect is not necessary to explain the data. Applying a threshold to a posterior probability (as in this paper) is a device to simplify the reporting of effects for which there is very strong evidence. The number of effects for which there was strong evidence may be small or large – and does not affect the posterior probability reporting threshold (or require a correction for the number of effects for which evidence was assessed).

whole model space consists of different combinations of forward, backward, and self-inhibitory connections in either temporal or frontal areas.

Following Bayesian model reduction, the posterior probabilities of these models accounting for the group difference effect (PScz > Con) are plotted in the middle. This shows that the PScz > Con effect is best described by Model 7, containing only the left and right IFG self-inhibitory connections. Instead of simply using the parameters estimated from the winning model (as there may not always be a clear winner), posterior parameter estimates are obtained by averaging over all models, weighted by their model evidence (Bayesian model averaging). Note that if there is no evidence for some models, parameters unique to those models will be redundant: these parameters are eliminated following Bayesian model reduction. Conversely, parameters that are present in all probable models are highly likely to explain the group difference effect. The posterior probabilities of parameters differing between groups are shown on the right (Figure S4A): only six parameters have non-zero probabilities of explaining the group difference effect, but only two of these are >0.95. These probabilities generate the Bayesian confidence intervals for the group difference plots (Figure 3F, in this case).

Figure S4B (left) shows the model space for the group differences in microcircuit parameters. The first six rows correspond to sp-sp self-inhibitory connections in areas from L A1 to R IFG; the next five rows containing white boxes correspond to connections constrained to be identical throughout the model. The (column-wise) model space consists of different combinations of connections to and from inhibitory interneurons, and of sp-sp self-inhibition at different levels of the model. The subsequent plots show the model and parameter posterior probabilities; the results of the analysis are below in Figure S4C (middle and right).

Data analysis and modelling: 40 Hz auditory steady state response

The timecourses of the 40 Hz ASSR waveforms in electrode Fz are plotted in Figure 4A: ~40 Hz oscillations are superimposed on more sustained baseline changes (known to occur during auditory click trains (58)). PScz and Rel response baselines diverge (*t*-tests at each timepoint) from Con around 250 ms, but we restricted our modelling to well-replicated group differences in ~40 Hz power. Each subjects' EEG data were transformed into measurements of phase and power in the frequency domain using a Morlet wavelet in spm_eeg_tf. For the

Fz sensor-level analyses, the spectra were normalised in order to assess the power at 40 Hz relative to the background 1/f slope. For normalisation, we again subtracted the 1/f gradient in log space (computed using robustfit in Matlab applied to the 10-30 Hz and 50-70 Hz ranges, on a subject-specific basis): example gradients of group averaged data from electrode Fz are shown in Figure S2E. γ peak frequency was the frequency in which the maximum (normalised) power within the 35-45 Hz range occurred (plotted in Figure 4B). The unnormalised group averaged time-frequency plots are shown in Figure S2F, and the normalised plots in Figure 4C. Permutation testing assessed whether the number of observed significant *t*-tests ($P<0.05$) for the Con v PScz, Con v Rel and PScz v Rel comparisons across all timepoints in the 30-55 Hz range shown was likely due to chance. There were no group differences in normalised power at other frequencies (*t*-tests at each timepoint and frequency, not shown).

The 40 Hz ASSR paradigm is known to activate primary auditory cortex (Heschl's gyrus), anterior to auditory evoked response sources (59,60). To obtain good priors for source localisation, we performed source localisation on all subjects in SPM12 using a minimum norm approach and focussing purely on the 37-43 Hz frequency band. Across all subjects, this generated three peaks of activation, either in or near right Heschl's gyrus and anterior to the MMN sources. The closest to Heschl's gyrus (A1) was [50 -12 4] (Figure 4D), which was used (along with [-50 -12 4]) as the seed for reconstruction of virtual electrode data from a broader band (35-50 Hz) for modelling in DCM.

The neuronal model for DCM had to be adjusted in order to model the high amplitude ~40 Hz peaks in the virtual electrode data from left and right A1 (dotted lines in Figure S3B). To fit these peaks we introduced a Gaussian 'bump' at 40 Hz (of width w≤4 Hz) into the background neuronal noise, to reflect the specific 40 Hz input (similar to a previous model (41) using a 10 Hz bump added to neuronal noise to simulate a thalamic input). As in the MMN analysis, we estimated group mean values for T (time constants) and also S (the slope of the sigmoid activation function) and J (the contribution of neuronal populations to the EEG signal) in an initial model inversion, and then used these as empirical priors (Figure S1A). These changes meant that sp and ii time constants were slightly shorter, there was increased gain in the transformation from membrane potential to firing rates, and that ss cells (which receive the 40 Hz thalamic drive) made more contribution to the measured EEG signal (respectively). We fixed T to its priors because without being fixed, they often became

biologically implausible. We did not fix D (delays) because these might have important effects on peak frequencies in this paradigm. As in the MMN analysis, we estimated six microcircuit (G) parameters but constrained all except sp-sp self-inhibition to be identical (within subjects) in A1 bilaterally. Last, we increased the precision of the data (hE), which was necessary to force the model to fit the unnatural peaks in the 35-50 Hz range. We attempted to fit a wider range of frequencies (15-50 Hz), but the model fitted the lower frequencies at the expense of the 40 Hz peak, ignoring the key data feature, so the narrower (γ-only) band of 35-50 Hz was chosen.

40 Hz ASSR model fitting was performed using the 'LFP' forward model in DCM for cross-spectral densities (CSD), without iterative fitting (because the increased data precision caused unrealistic parameter changes). Model comparison between the model with new priors for T, S and J and models with previous versions of each of these priors was performed, to ensure the new priors improved the model (Figure 4E, left). The comparison also included a model with a narrower 40 Hz bump (w=1 Hz). The first ten subjects' data (dotted lines) and model fits (thick lines) for the best model are shown in Figure S3B. The model fits reasonably well – it often cannot capture the full amplitude of the ~40 Hz peak, but usually has its peak at the right frequency: one exception is subject 0254 (bottom right), whose unusual peaks at 45 Hz are too far from 40 Hz for the model to accommodate. R^2 values for the winning model are shown in Figure 4E (right), and group differences between Con and PScz or Rel in their R^2 values were also assessed (Figure S3C). R^2 values were more variable in the 40 Hz ASSR than the other paradigms, because in the 40 Hz ASSR the model was trying to fit a small data feature very precisely. 19 subjects failed model inversion (added to 12 who failed preprocessing: Table S1).

The model space used for the analysis of A1 microcircuit (G) parameters within PEB is shown in Figure S5A (first). The first two rows denote sp-sp self-inhibition on the left and right, and the next five rows containing white boxes denote the remaining microcircuit parameters which were identical bilaterally. The (column-wise) model space is constituted by different combinations of sp and ii self-inhibitory parameters and ii input and output parameters. Following Bayesian model reduction, no model of the group differences dominates (middle), however all models with any evidence for them contain sp-sp self-inhibition parameters, so the posterior probability (after Bayesian model averaging) that these parameters contribute to the group difference is very high (right).

Data analysis and modelling: resting state fMRI

We restricted the rsfMRI analysis to being directly comparable to the MMN and 40 Hz ASSR analyses. We therefore chose to analyse effective connectivity in exactly the same network involved in the MMN (and 40 Hz ASSR, in part), i.e. bilateral A1, STG and IFG. The sources used for the MMN lie within areas A1, A4 and 44 of the Glasser parcellation (25) (Figure 1), so these were chosen to be the nodes of the network. We did not use subject-specific sources from the MMN source reconstruction, because the MMN sources are well-established, and if a subject's EEG source reconstruction lay outside these areas, it is arguably more likely that their EEG source reconstruction was inaccurate than that a different cortical network activated in their MMN. Thus, using the same (individually parcellated) rsfMRI sources for each subject took account of individual variation in anatomy whilst ensuring the rsfMRI analysis was robust and reproducible.

For the DCM analysis we used the movement-scrubbed rsfMRI data with global signal regression. Although global signal likely contains neuronal signal that is relevant to schizophrenia (61), we removed it in order to reduce global physiological confounds as much as possible. No subjects were excluded due to low SNR, although doing so (for the ten subjects with SNR<25) did not change any of the results. Model structure was assumed to be as it was in the MMN analysis (Figure 1), with forward and backward connections linking adjacent hierarchical levels only, and lateral connections (not shown) linking bilateral areas at the same level; forward, backward and self-inhibitory connections could differ between groups.

Spectral DCM for rsfMRI was used to estimate effective connectivity. Spectral DCM estimates the amplitude and exponent parameters of the low frequency (<0.1 Hz) cross spectra of endogenous neuronal fluctuations, and thus the covariance of the underlying hidden states (i.e. neuronal activity in each node) (62). The advantage of this power-law model is that it is deterministic – i.e. less computationally demanding – whilst accommodating stochastic fluctuations in neural states. In this model, the units of connectivity are Hz, i.e. stronger connectivity produces a faster rate of responding in a target region. The dynamics of the neuronal model are mapped to the fMRI signal via a haemodynamic forward model (63). No model comparison was performed prior to PEB

analysis in order to match the same model structure used in the MMN analysis, for comparative purposes, and priors did not need to be adjusted to improve model fit. The R^2 values (and their differences between groups) are shown in Figure S3C.

Again, PEB was used to analyse group differences in parameters. The model space for the PScz > Con analysis is shown in Figure S6A (left). It comprises different combinations of forward, backward and self-inhibitory connections. There is no clear winning model (middle) but all likely models contained bilateral IFG self-inhibitory connections, hence these connections are inferred to be highly likely to explain group differences (right).

For comparative purposes, in addition to the spectral DCM analysis, we performed a standard functional connectivity analysis (Figure S6B), i.e. computing the Pearson (zero lag) correlation between two parcellations' timeseries. We also computed their (zero lag) covariance (Figure S6C), because comparing correlations in Con and PScz can be problematic as their variances may differ (61), and the corresponding zero lag covariance in the neuronal states estimated by DCM (Figure S6D). To see whether spectral DCM self-inhibition parameters might be recapitulated in basic data features, we also assessed group differences in the coefficient of variation (standard deviation normalised by the mean, computed using an unbiased method) of BOLD signal in each node (Figure S6E), given that greater self-inhibition would be expected to relate to lower variance in a node (64).

It is clear from Figures 5A and S6B-E that one cannot reliably associate effective connectivity with functional connectivity or BOLD fluctuations. Reduced functional connectivity between A1 and STG in PScz (Figure S6B and elsewhere (65)) is less robust if covariance is used instead (an issue highlighted previously (61)). Conversely, increased IFG self-inhibition in PScz (Figure 5A) is not apparent in the BOLD standard deviations in IFG (Figure S6E), although reduced BOLD fluctuations in PScz have been reported in other areas (64). Measures of functional connectivity differ from effective connectivity in several important ways: i) spectral DCM also estimates haemodynamic and neuronal/measurement noise parameters – which may contribute to group differences in functional connectivity e.g. if one group is older (66) or taking (antipsychotic) medication, or if one group moves more (respectively); ii) spectral DCM connectivity includes non-zero lags, which indicate direction of connectivity (62,67); iii) and – most importantly – spectral DCM also estimates self-inhibition within cortical regions.

We also performed the same DCM and PEB analyses without global signal regression, to ascertain the effects of this preprocessing step on the results (Figure S7).

Data analysis and modelling: parameter sensitivity and cross-paradigm analysis

Numerous studies have shown that DCM can accurately recover group-specific parameter changes when the ground truth is known: for example, inferring the known physiological effects of isoflurane anaesthesia from rodent cross-spectral data (68), inferring antiNMDA receptor antibodies have specific effects on NMDA receptor parameters from human rsEEG (36), and reliably recovering parameter values (using DCM and PEB) from simulated MMN EEG data (52) and from simulated rsfMRI data (62).

Nevertheless, we performed parameter sensitivity analyses on the six estimated microcircuit (G) parameters to address two potential weaknesses in the MMN and 40 Hz ASSR analyses. In the MMN analysis, constraining condition-specific (mismatch) effects to only sp-sp self-inhibition amongst the G parameters – along with extrinsic connections – might miss such effects in other G parameters. In the 40 Hz ASSR analysis, the data were afforded high precision during model fitting in order to force the model to fit the unnatural 40 Hz peak: this may lead to overfitting of other data features, however, and spurious results. We therefore selected two subjects from each paradigm with EEG responses typical of the group average. We simulated virtual electrode ('LFP') data from a single cortical area using spm_induced_optimise and either spm_gen_erp (for the MMN) or spm_csd_mtf (for the 40 Hz ASSR). In each case we used these subjects' posterior G parameter estimates, varying each G parameter by ±30% in turn; in the MMN, we also fixed delays (D) and time constants (T) to the values used in Figure S1A. The simulated data are shown in Figure S8, with the key data features that the parameters showing group differences ought to explain circled in red.

We used the PEB framework to assess whether the abnormal parameters in the PScz group related to each other across modalities, i.e. across EEG and fMRI. If so, this would support the use of multiple paradigms in combination to estimate these 'biomarker' parameters. We selected the left and right IFG self-inhibition parameters from the MMN and left and right A1 self-inhibition parameters from the 40 Hz ASSR, and their counterparts from the rsfMRI. For

each of the four rsfMRI parameters separately, we used the PEB framework to reveal which of all MMN or 40 Hz ASSR parameters related to it. Thus the posterior expectation and covariance in the EEG parameters, but only the expected value of the rsfMRI parameter, was used by the PEB model. (We reasoned that the rsfMRI parameters were likely estimated with greater confidence, given their simpler models and more consistently high R^2 values compared with the EEG models (Figure S3C), hence it would be better to include the EEG parameters' covariance in the PEB model.) Two of the four analyses yielded significant results: R IFG self-inhibition across rsfMRI and MMN (Figure S9A), and R A1 self-inhibition across rsfMRI and 40 Hz ASSR (Figure S9B).

For comparative purposes, we also performed classical analyses of the relationships between the same rsfMRI and EEG parameters. The Pearson correlations between the rsfMRI and 40 Hz ASSR or MMN self-inhibition parameters are shown in Figure S9C. Both EEG paradigms – especially the MMN – show some clustering of the parameters around their prior values (0 in the 40 Hz ASSR, and 0.5 in the MMN). Posterior parameter values that are further from the priors are typically estimated with greater confidence. The advantage of the PEB framework is that it will (optimally) upweight the contribution of these more confident parameters to group-level inference.

Supplementary Results

Excluding benzodiazepine effects on rsEEG

The group differences in rsEEG power were unlikely to be driven by participants taking benzodiazepines, as daily use of these drugs was an exclusion criterion, and removal of these subjects (5 Con, 16 PScz) did not substantially alter the effects, although Bonferroni-correction now shifted the gamma difference to trend-level significance. Compared with Con (n=102), PScz (n=92) had increased θ (Z=2.49, P(corr)=0.039), decreased β (Z=-2.55, P(corr)=0.032), and increased γ (Z=2.34, P(corr)=0.059). In awake humans, benzodiazepines typically cause increased δ, *decreased* θ and α, and *increased* β power (69,70): a different pattern to that observed in PScz.

Many rsfMRI results were lost if global signal regression was not performed

We repeated the rsfMRI analyses without global signal regression (Figures S7A-D). Many results changed, with much greater between subject variability in parameters (the PScz > Con contrast, Figure S7A) or the loss of significant effects (Figures S7B and D). Although global signal regression (GSR) removes physiological 'noise', the global signal has greater power in PScz, which may have a neuronal component (61). Here, GSR strengthened existing but weak effects in both PScz and Con (Figures 5C-E and S7C-E), and converted reduced forward connectivity into IFG (amidst a large amount of between-subject parameter variability, Figure S7A) to reduced synaptic gain within IFG. Given this, and given GSR in PScz affords a relatively uniform transform of the data (61), the results with GSR are probably more reliable, especially given their consilience with the EEG results (Figure 6).

Relationships between rsfMRI and EEG data features and self-inhibition parameters, and the possibility of 'model-based biomarkers'

We assessed relationships of rsfMRI data features to MMN amplitude and 40 Hz ASSR maximum γ power. In Scz, MMN (absolute) amplitude correlated with the coefficient of variation (the standard deviation normalised by the mean) of BOLD in almost every node of the MMN network: L A1 (r=0.49, P=0.00003), R A1 (r=0.42, P=0.0005), L STG (r=0.27, P=0.027), R STG (r=0.28, P=0.023), L IFG (r=0.23, P=0.07) and R IFG (r=0.33, P=0.008). Only bilateral A1 and R IFG would survive correction for 6 comparisons, however. None of these relationships were significant in Con (all P>0.2) or Rel (all P>0.1). There were no

relationships between 40 Hz ASSR γ power and rsfMRI BOLD coefficient of variation in A1 in any group (all *P*>0.07).

Finally, in *post hoc* analyses, we asked whether self-inhibition parameters – that were altered in PScz – had predictive validity across paradigms, thus licensing their use as 'model-based biomarkers'. We used PEB to assess relationships in PScz between self-inhibition parameters in IFG in the MMN, or in A1 in the 40 Hz ASSR, and their corresponding self-inhibition parameters in the rsfMRI, in participants whose EEG and rsfMRI were <100 days apart (MMN n=37, 40Hz ASSR n=41), as some participants underwent EEG and rsfMRI many months or even years apart. Within PScz, there was evidence of associations between self-inhibition in R IFG in the MMN and rsfMRI (*P*>0.95; Figure S9A) and in R A1 in the 40 Hz ASSR and rsfMRI (*P*>0.95; Figure S9B). However, there were no such relationships in the left hemisphere or in Con.

To explore these relationships further we assessed their Pearson correlations (Figure S9C). None were significant (all *P*>0.05, uncorrected), and posterior parameter estimates clustered around the priors (of 0 in the 40 Hz ASSR, and 0.5 in the MMN), especially in the MMN, indicating the PEB results were driven by the subset of parameters estimated with sufficient efficiency to be drawn away from the prior.

This clustering of EEG estimates around their prior values must be finessed (by using more informative data or paradigm design) to assess cross-paradigm self-inhibition reliability in all participants. If parameters show cross-paradigm validity, hierarchical PEB can be used to estimate them from multiple paradigms simultaneously (51), and thereby provide model-based biomarkers for psychosis.

Reduced self-inhibition relates to Digit Symbol performance in Con across three tasks

We also assessed relationships of DCM parameters to a cognitive measure: Digit Symbol task performance (Figure S10). In the MMN, across both conditions, reduced self-inhibition in bilateral IFG in Con was associated with greater information processing speed (i.e. Digit Symbol Substitution Task score; *P*>0.99 and *P*>0.95, Figure S10A), but there were no such relationships in PScz (all *P*<0.95).

In the 40 Hz ASSR, in Con and PScz, Digit Symbol performance was associated with reduced pyramidal self-inhibition in right and left A1 respectively (both $P>0.99$) and reduced deep pyramidal to inhibitory interneuron (dp-ii) connectivity ($P>0.99$; Figure S10B).

In the rsfMRI, Digit Symbol performance was associated with decreased R IFG self-inhibition in Con ($P>0.99$; Figure S10C) but not in PScz, who instead showed decreased L A1-STG and increased L STG-A1 connectivity (both $P>0.99$, not shown).

Thus, processing speed (i.e. Digit Symbol performance) in Con was associated with disinhibition (or increased synaptic gain) across three paradigms, which speaks to the predictive validity of the synaptic biomarkers furnished by DCM. This possible relationship between cognitive performance and synaptic gain complements a recent rsfMRI DCM study of healthy adults (n=602) that found fluid intelligence was related to decreased self-inhibition in dorsal attention and salience networks (66). R IFG self-inhibition's relationship to Digit Symbol performance (in Con) also mirrors the finding that the global functional connectivity of a nearby region of left lateral prefrontal cortex relates to fluid intelligence (71).

In PScz, however, self-inhibition and cognition were only associated in R A1 (in the 40 Hz ASSR). Interestingly, a rsEEG DCM study in Down syndrome (n=36) found intelligence was associated with self-inhibition in V1 (72). Why cognition relates to self-inhibition in primary sensory regions in these groups is unclear: it may be estimated more efficiently there.

Supplementary References

1. First MB, Williams JB, Spitzer RL, Gibbon M (2007): Structured clinical interview for DSM-IV-TR Axis I disorders, clinical trials version (SCID-CT). *New York: Biometrics Research, New York State Psychiatric Institute*.
2. Wechsler D (1997): *WAIS-III: Administration and Scoring Manual: Wechsler Adult Intelligence Scale.* Psychological Corporation.
3. Puvvada KC, Summerfelt A, Du X, Krishna N, Kochunov P, Rowland LM, *et al.* (2018): Delta Vs Gamma Auditory Steady State Synchrony in Schizophrenia. *Schizophr Bull* 44: 378–387.
4. Dickinson D, Ramsey ME, Gold JM (2007): Overlooking the obvious: a meta-analytic comparison of digit symbol coding tasks and other cognitive measures in schizophrenia. *Arch Gen Psychiatry* 64: 532–542.
5. Rowland LM, Summerfelt A, Wijtenburg SA, Du X, Chiappelli JJ, Krishna N, *et al.* (2016): Frontal Glutamate and γ-Aminobutyric Acid Levels and Their Associations With Mismatch Negativity and Digit Sequencing Task Performance in Schizophrenia. *JAMA Psychiatry* 73: 166–174.
6. Delorme A, Makeig S (2004): EEGLAB: an open source toolbox for analysis of single-trial EEG dynamics including independent component analysis. *J Neurosci Methods* 134: 9–21.
7. Winkler I, Haufe S, Tangermann M (2011): Automatic classification of artifactual ICA-components for artifact removal in EEG signals. *Behav Brain Funct* 7: 30.
8. Litvak V, Mattout J, Kiebel S, Phillips C, Henson R, Kilner J, *et al.* (2011): EEG and MEG data analysis in SPM8. *Comput Intell Neurosci* 2011: 852961.

9. Ji JL, Diehl C, Schleifer C, Tamminga CA, Keshavan MS, Sweeney JA, *et al.* (2019): Schizophrenia Exhibits Bi-directional Brain-Wide Alterations in Cortico-Striato-Cerebellar Circuits. *Cereb Cortex* 29: 4463–4487.

10. Jenkinson M, Bannister P, Brady M, Smith S (2002): Improved optimization for the robust and accurate linear registration and motion correction of brain images. *Neuroimage* 17: 825–841.

11. Reuter M, Schmansky NJ, Rosas HD, Fischl B (2012): Within-subject template estimation for unbiased longitudinal image analysis. *Neuroimage* 61: 1402–1418.

12. Glasser MF, Sotiropoulos SN, Wilson JA, Coalson TS, Fischl B, Andersson JL, *et al.* (2013): The minimal preprocessing pipelines for the Human Connectome Project. *Neuroimage* 80: 105–124.

13. Anticevic A, Gancsos M, Murray JD, Repovs G, Driesen NR, Ennis DJ, *et al.* (2012): NMDA receptor function in large-scale anticorrelated neural systems with implications for cognition and schizophrenia. *Proc Natl Acad Sci USA* 109: 16720–16725.

14. Anticevic A, Brumbaugh MS, Winkler AM, Lombardo LE, Barrett J, Corlett PR, *et al.* (2013): Global prefrontal and fronto-amygdala dysconnectivity in bipolar I disorder with psychosis history. *Biol Psychiatry* 73: 565–573.

15. Power JD, Barnes KA, Snyder AZ, Schlaggar BL, Petersen SE (2012): Spurious but systematic correlations in functional connectivity MRI networks arise from subject motion. *Neuroimage* 59: 2142–2154.

16. Power JD, Barnes KA, Snyder AZ, Schlaggar BL, Petersen SE (2013): Steps toward optimizing motion artifact removal in functional connectivity MRI; a reply to Carp. *Neuroimage* 76: 439–441.

17. Biswal BB, Mennes M, Zuo X-N, Gohel S, Kelly C, Smith SM, *et al.* (2010): Toward discovery science of human brain function. *Proc Natl Acad Sci USA* 107: 4734–4739.

18. Fischl B, Salat DH, Busa E, Albert M, Dieterich M, Haselgrove C, *et al.* (2002): Whole brain segmentation: automated labeling of neuroanatomical structures in the human brain. *Neuron* 33: 341–355.

19. Repovs G, Csernansky JG, Barch DM (2011): Brain network connectivity in individuals with schizophrenia and their siblings. *Biol Psychiatry* 69: 967–973.

20. Power JD, Plitt M, Laumann TO, Martin A (2017): Sources and implications of whole-brain fMRI signals in humans. *Neuroimage* 146: 609–625.

21. Yang GJ, Murray JD, Glasser M, Pearlson GD, Krystal JH, Schleifer C, *et al.* (2017): Altered Global Signal Topography in Schizophrenia. *Cereb Cortex* 27: 5156–5169.

22. Glasser MF, Coalson TS, Bijsterbosch JD, Harrison SJ, Harms MP, Anticevic A, *et al.* (2018): Using temporal ICA to selectively remove global noise while preserving global signal in functional MRI data. *Neuroimage* 181: 692–717.

23. Aquino KM, Fulcher BD, Parkes L, Sabaroedin K, Fornito A (2020): Identifying and removing widespread signal deflections from fMRI data: Rethinking the global signal regression problem. *Neuroimage* 212: 116614.

24. Yang GJ, Murray JD, Wang X-J, Glahn DC, Pearlson GD, Repovs G, *et al.* (2016): Functional hierarchy underlies preferential connectivity disturbances in schizophrenia. *Proc Natl Acad Sci USA* 113: E219-228.

25. Glasser MF, Coalson TS, Robinson EC, Hacker CD, Harwell J, Yacoub E, *et al.* (2016): A multi-modal parcellation of human cerebral cortex. *Nature* 536: 171–178.

26. Moran R, Pinotsis DA, Friston K (2013): Neural masses and fields in dynamic causal modeling. *Front Comput Neurosci* 7: 57.

27. Pinotsis DA, Schwarzkopf DS, Litvak V, Rees G, Barnes G, Friston KJ (2013): Dynamic causal modelling of lateral interactions in the visual cortex. *Neuroimage* 66: 563–576.

28. Jansen BH, Rit VG (1995): Electroencephalogram and visual evoked potential generation in a mathematical model of coupled cortical columns. *Biol Cybern* 73: 357–366.

29. Bastos AM, Usrey WM, Adams RA, Mangun GR, Fries P, Friston KJ (2012): Canonical Microcircuits for Predictive Coding. *Neuron* 76: 695–711.

30. Auksztulewicz R, Friston K (2015): Attentional Enhancement of Auditory Mismatch Responses: a DCM/MEG Study. *Cereb Cortex* 25: 4273–4283.

31. Deleuze C, Pazienti A, Bacci A (2014): Autaptic self-inhibition of cortical GABAergic neurons: synaptic narcissism or useful introspection? *Curr Opin Neurobiol* 26: 64–71.

32. Dienel SJ, Lewis DA (2019): Alterations in cortical interneurons and cognitive function in schizophrenia. *Neurobiol Dis* 131: 104208.

33. Tsubomoto M, Kawabata R, Zhu X, Minabe Y, Chen K, Lewis DA, Hashimoto T (2019): Expression of Transcripts Selective for GABA Neuron Subpopulations across the Cortical Visuospatial Working Memory Network in the Healthy State and Schizophrenia. *Cereb Cortex* 29: 3540–3550.

34. Shaw AD, Moran RJ, Muthukumaraswamy SD, Brealy J, Linden DE, Friston KJ, Singh KD (2017): Neurophysiologically-informed markers of individual variability and pharmacological manipulation of human cortical gamma. *Neuroimage* 161: 19–31.

35. Adams NE, Hughes LE, Phillips HN, Shaw AD, Murley AG, Nesbitt D, *et al.* (2020): GABA-ergic Dynamics in Human Frontotemporal Networks Confirmed by Pharmaco-Magnetoencephalography. *J Neurosci* 40: 1640–1649.

36. Symmonds M, Moran CH, Leite MI, Buckley C, Irani SR, Stephan KE, *et al.* (2018): Ion channels in EEG: isolating channel dysfunction in NMDA receptor antibody encephalitis. *Brain* 141: 1691–1702.

37. Bastos AM, Briggs F, Alitto HJ, Mangun GR, Usrey WM (2014): Simultaneous Recordings from the Primary Visual Cortex and Lateral Geniculate Nucleus Reveal Rhythmic Interactions and a Cortical Source for Gamma-Band Oscillations. *J Neurosci* 34: 7639–7644.

38. Roopun AK, Middleton SJ, Cunningham MO, LeBeau FEN, Bibbig A, Whittington MA, Traub RD (2006): A beta2-frequency (20-30 Hz) oscillation in nonsynaptic networks of somatosensory cortex. *Proc Natl Acad Sci USA* 103: 15646–15650.

39. Kramer MA, Roopun AK, Carracedo LM, Traub RD, Whittington MA, Kopell NJ (2008): Rhythm Generation through Period Concatenation in Rat Somatosensory Cortex. *PLOS Computational Biology* 4: e1000169.

40. Vijayan S, Kopell NJ (2012): Thalamic model of awake alpha oscillations and implications for stimulus processing. *PNAS* 109: 18553–18558.

41. Pinotsis DA, Geerts JP, Pinto L, FitzGerald THB, Litvak V, Auksztulewicz R, Friston KJ (2017): Linking canonical microcircuits and neuronal activity: Dynamic causal modelling of laminar recordings. *Neuroimage* 146: 355–366.

42. Umbricht D, Krljes S (2005): Mismatch negativity in schizophrenia: a meta-analysis. *Schizophrenia Research* 76: 1–23.

43. Garrido MI, Kilner JM, Stephan KE, Friston KJ (2009): The mismatch negativity: a review of underlying mechanisms. *Clin Neurophysiol* 120: 453–463.

44. Fish KN, Rocco BR, DeDionisio AM, Dienel SJ, Sweet RA, Lewis DA (2021): Altered Parvalbumin Basket Cell Terminals in the Cortical Visuospatial Working Memory Network in Schizophrenia. *Biol Psychiatry*. https://doi.org/10.1016/j.biopsych.2021.02.009

45. Burt JB, Demirtaş M, Eckner WJ, Navejar NM, Ji JL, Martin WJ, *et al.* (2018): Hierarchy of transcriptomic specialization across human cortex captured by structural neuroimaging topography. *Nat Neurosci* 21: 1251–1259.

46. Weickert CS, Fung SJ, Catts VS, Schofield PR, Allen KM, Moore LT, *et al.* (2013): Molecular evidence of N-methyl-D-aspartate receptor hypofunction in schizophrenia. *Mol Psychiatry* 18: 1185–1192.

47. Schmidt A, Bachmann R, Kometer M, Csomor PA, Stephan KE, Seifritz E, Vollenweider FX (2012): Mismatch Negativity Encoding of Prediction Errors Predicts S-ketamine-Induced Cognitive Impairments. *Neuropsychopharmacology* 37: 865–875.

48. Moran RJ, Campo P, Symmonds M, Stephan KE, Dolan RJ, Friston KJ (2013): Free energy, precision and learning: the role of cholinergic neuromodulation. *J Neurosci* 33: 8227–8236.

49. Fastenrath M, Friston KJ, Kiebel SJ (2009): Dynamical causal modelling for M/EEG: spatial and temporal symmetry constraints. *Neuroimage* 44: 154–163.

50. Fuchs M, Wagner M, Kastner J (2001): Boundary element method volume conductor models for EEG source reconstruction. *Clin Neurophysiol* 112: 1400–1407.

51. Friston KJ, Litvak V, Oswal A, Razi A, Stephan KE, van Wijk BCM, *et al.* (2016): Bayesian model reduction and empirical Bayes for group (DCM) studies. *Neuroimage* 128: 413–431.

52. Friston K, Zeidman P, Litvak V (2015): Empirical Bayes for DCM: A Group Inversion Scheme. *Front Syst Neurosci* 9: 164.

53. Zeidman P, Jafarian A, Seghier ML, Litvak V, Cagnan H, Price CJ, Friston KJ (2019): A guide to group effective connectivity analysis, part 2: Second level analysis with PEB. *Neuroimage* 200: 12–25.

54. Stephan KE, Penny WD, Daunizeau J, Moran RJ, Friston KJ (2009): Bayesian model selection for group studies. *Neuroimage* 46: 1004–1017.

55. Rigoux L, Stephan KE, Friston KJ, Daunizeau J (2014): Bayesian model selection for group studies - revisited. *Neuroimage* 84: 971–985.

56. Pinotsis DA, Perry G, Litvak V, Singh KD, Friston KJ (2016): Intersubject variability and induced gamma in the visual cortex: DCM with empirical Bayes and neural fields. *Hum Brain Mapp* 37: 4597–4614.

57. Friston KJ, Penny W (2011): Post hoc Bayesian model selection. *Neuroimage* 56: 2089–2099.

58. Gutschalk A, Patterson RD, Scherg M, Uppenkamp S, Rupp A (2004): Temporal dynamics of pitch in human auditory cortex. *Neuroimage* 22: 755–766.

59. Ross B, Draganova R, Picton TW, Pantev C (2003): Frequency specificity of 40-Hz auditory steady-state responses. *Hear Res* 186: 57–68.

60. Sivarao DV (2015): The 40-Hz auditory steady-state response: a selective biomarker for cortical NMDA function. *Ann N Y Acad Sci* 1344: 27–36.

61. Yang GJ, Murray JD, Repovs G, Cole MW, Savic A, Glasser MF, *et al.* (2014): Altered global brain signal in schizophrenia. *Proc Natl Acad Sci USA* 111: 7438–7443.

62. Razi A, Kahan J, Rees G, Friston KJ (2015): Construct validation of a DCM for resting state fMRI. *Neuroimage* 106: 1–14.

63. Friston KJ, Mechelli A, Turner R, Price CJ (2000): Nonlinear responses in fMRI: the Balloon model, Volterra kernels, and other hemodynamics. *Neuroimage* 12: 466–477.

64. Kaufmann T, Skåtun KC, Alnæs D, Doan NT, Duff EP, Tønnesen S, *et al.* (2015): Disintegration of Sensorimotor Brain Networks in Schizophrenia. *Schizophr Bull* 41: 1326–1335.

65. Lee M, Sehatpour P, Hoptman MJ, Lakatos P, Dias EC, Kantrowitz JT, *et al.* (2017): Neural mechanisms of mismatch negativity dysfunction in schizophrenia. *Mol Psychiatry* 22: 1585–1593.

66. Tsvetanov KA, Henson RNA, Tyler LK, Razi A, Geerligs L, Ham TE, *et al.* (2016): Extrinsic and Intrinsic Brain Network Connectivity Maintains Cognition across the Lifespan Despite Accelerated Decay of Regional Brain Activation. *J Neurosci* 36: 3115–3126.

67. Mitra A, Snyder AZ, Blazey T, Raichle ME (2015): Lag threads organize the brain's intrinsic activity. *Proc Natl Acad Sci USA* 112: E2235-2244.

68. Moran RJ, Jung F, Kumagai T, Endepols H, Graf R, Dolan RJ, *et al.* (2011): Dynamic causal models and physiological inference: a validation study using isoflurane anaesthesia in rodents. *PLoS One* 6: e22790.

69. Saletu B, Anderer P, Saletu-Zyhlarz GM (2006): EEG topography and tomography (LORETA) in the classification and evaluation of the pharmacodynamics of psychotropic drugs. *Clin EEG Neurosci* 37: 66–80.

70. Gilles C, Luthringer R (2007): Pharmacological models in healthy volunteers: their use in the clinical development of psychotropic drugs. *J Psychopharmacol* 21: 272–282.

71. Cole MW, Yarkoni T, Repovs G, Anticevic A, Braver TS (2012): Global connectivity of prefrontal cortex predicts cognitive control and intelligence. *J Neurosci* 32: 8988–8999.

72. Hamburg S, Rosch R, Startin CM, Friston KJ, Strydom A (2019): Dynamic Causal Modeling of the Relationship between Cognition and Theta-alpha Oscillations in Adults with Down Syndrome. *Cereb Cortex* 29: 2279–2290.

A

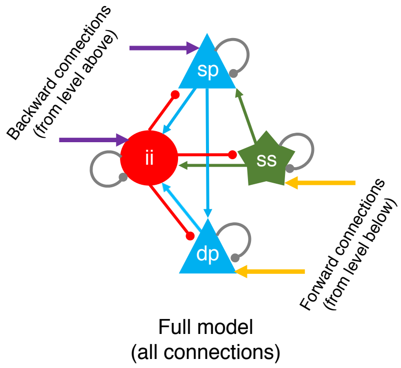

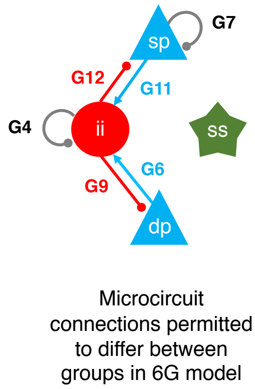

Para meter	Description	rsEEG prior mean	MMN prior mean	MMN prior precision	40 Hz ASSR prior mean	40 Hz ASSR prior precision
G1	ss self-inhibition (gain)	800	800	0	800	0
G3	ii connectivity to ss	800	800	0	800	0
**G4**	ii self-inhibition (gain)	800	240	1/32	800	1/32
G5	ss connectivity to ii	800	800	0	800	0
G6	dp connectivity to ii	400	270	1/32	400	1/32
**G7**	sp self-inhibition (gain)	800	1300	1/32	800	1/32
G8	ss connectivity to sp	800	800	0	800	0
G9	ii connectivity to dp	400	730	1/32	400	1/32
G10	dp self-inhibition (gain)	200	200	0	200	0
G11	sp connectivity to ii	800	1800	1/32	800	1/32
G12	ii connectivity to sp	800	980	1/32	800	1/32
G13	sp connectivity to dp	800	800	0	800	0
T1	Time constant: ss	2 ms	2 ms	0	5 ms	0
T2	Time constant: sp	2 ms	2.5 ms	0	1.5 ms	0
T3	Time constant: ii	16 ms	26 ms	0	8 ms	0
T4	Time constant: dp	28 ms	60 ms	0	38 ms	0
D1	Delay constant: intrinsic	1 ms	0.8 ms	0	1 ms	1/64
D2	Delay constant: extrinsic	8 ms	8 ms	0	8 ms	1/64
S	Slope of sigmoid activation function	1	1	1/64	1.6	1/64
w	Maximum width of ~40 Hz Gaussian in spectrum	n/a	n/a	n/a	4 Hz	0
J	Contributing states to MEEG [ss sp ii dp]	n/a	[0 1 0 0]	[1/32 0 0 1/32]	[0.3 1 0 0]	[1/32 0 0 1/32]
hE	Data precision	n/a	6	0.5^5	16	0.5^{14}

B

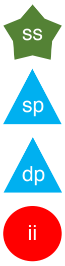

$$\ddot{V}_{ss} + 2\kappa_{ss}\dot{V}_{ss} + \kappa_{ss}^2 V_{ss} = \kappa_{ss}\left(A_F \cdot \sigma(V_{sp}) - g_{ii\to ss}\cdot\sigma(V_{ii}) - g_{ss\to ss}\cdot\sigma(V_{ss}) + U\right)$$

$$\ddot{V}_{sp} + 2\kappa_{sp}\dot{V}_{sp} + \kappa_{sp}^2 V_{sp} = \kappa_{sp}\left(-A_B \cdot \sigma(V_{dp}) + g_{ss\to sp}\cdot\sigma(V_{ss}) - g_{ii\to sp}\cdot\sigma(V_{ii}) - g_{sp\to sp}\cdot\sigma(V_{sp})\right)$$

$$\ddot{V}_{dp} + 2\kappa_{dp}\dot{V}_{dp} + \kappa_{dp}^2 V_{dp} = \kappa_{dp}\left(A_F \cdot \sigma(V_{sp}) + g_{sp\to dp}\cdot\sigma(V_{sp}) - g_{ii\to dp}\cdot\sigma(V_{ii}) - g_{dp\to dp}\cdot\sigma(V_{dp})\right)$$

$$\ddot{V}_{ii} + 2\kappa_{ii}\dot{V}_{ii} + \kappa_{ii}^2 V_{ii} = \kappa_{ii}\left(-A_B \cdot \sigma(V_{dp}) + g_{ss\to ii}\cdot\sigma(V_{ss}) + g_{sp\to ii}\cdot\sigma(V_{sp}) + g_{dp\to ii}\cdot\sigma(V_{dp}) - g_{ii\to ii}\cdot\sigma(V_{ii})\right)$$

Where

$$\kappa = 1/T$$

$$\dot{V} = I$$

$$V_{out} = g \otimes \sigma, \quad g(t) = \begin{cases} G\kappa t \cdot \exp(-\kappa t)\,; t \geq 0 \\ 0\,; t < 0 \end{cases}$$

$$\sigma = \frac{1}{1 + \exp\left(-S \cdot V_{in} + V_0\right)}$$

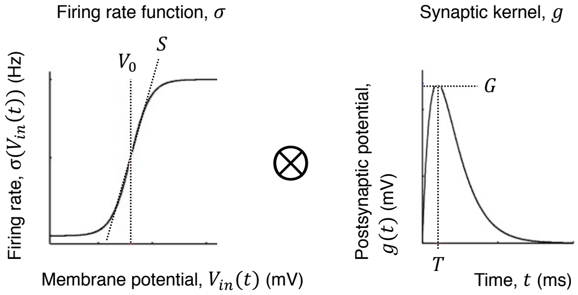

Figure S1 – DCM parameters and model for EEG paradigms

A – Left: The top panel shows the cortical microcircuit model used in the EEG paradigms. The four neuronal populations are spiny stellate cells (ss), superficial and deep pyramidal

cells (sp and dp) and inhibitory interneurons (ii). Each send either excitatory or inhibitory connections to other neuronal populations (coloured connections: arrows are excitatory, balls are inhibitory), and also have self-inhibitory connections (grey) that parameterise their responsiveness to their input, i.e. synaptic gain. The bottom panel shows the subset of connections in this microcircuit that were estimated in the MMN and 40 Hz ASSR paradigms, and could thus differ between groups.
Right: The table contains the parameters (prior expectation and precision or inverse variance) of the EEG DCMs. All parameters except the final two rows pertain to the neuronal model; the final two pertain to the forward model (i.e. how the neuronal model generates the EEG signal). G denote 'intrinsic' connectivities, i.e. connection strengths within the microcircuit, in arbitrary units. T denote time constants in each neuronal population, and D denote transmission delays between populations (intrinsic) or between areas (extrinsic). w was a new parameter used to model the ~40 Hz peak in the power spectrum introduced by the click train in the 40 Hz ASSR paradigm. J, the states contributing to the EEG signal, assumes that sp cells make the biggest contribution, but also permits ss and dp cells to do so. DCM was only used to simulate rsEEG power spectra, hence no forward model parameters (or parameter precisions) were used. The neuronal model parameter settings were unchanged from their standard values. In the MMN analysis, group mean T and D parameters were estimated and then fixed to these values; six G parameters were also estimated and used as priors. Standard forward model settings were used. In the 40 Hz ASSR, T were again fixed to the group mean values, and group mean values were used as priors for S, w and J. The data precision was increased to ensure the model fitted the unnatural 40 Hz peak.
B – The equations describing dynamics within the canonical microcircuit illustrated above. Terms are coloured according to the connections illustrated in the full model above. Changes in membrane currents $\dot{I} = \ddot{V}$ are a function of membrane rate constants $\kappa = 1/T$ (time constants T are given in the table above), afferent forward and backward connections A_F, A_B (in the MMN model only), synaptic kernels g, a sigmoid firing rate function σ (parameterised by S), and thalamic input U (to only the bottom – i.e. primary sensory – nodes in the hierarchy). Synaptic kernels g (parameterised by connectivity or maximum postsynaptic potential G and time constant T) are convolved with presynaptic firing input (a function σ of presynaptic membrane depolarisation V_{in}), to produce postsynaptic depolarisation V_{out}.

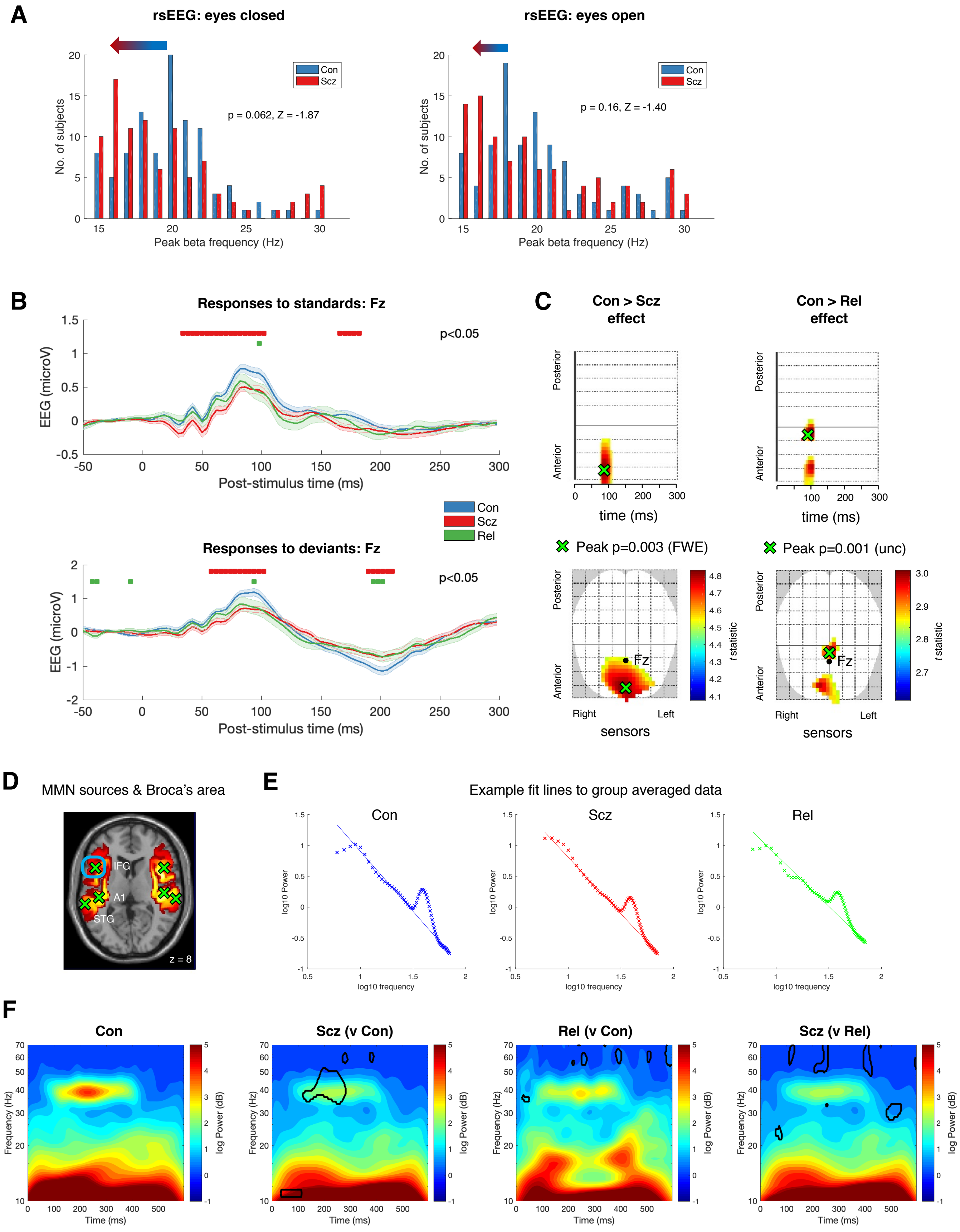

Figure S2 - Additional rsEEG, MMN & 40 Hz ASSR (not normalized) results

A – These histograms depict the numerical (but not significant) reduction in rsEEG peak β frequency in PScz (n=95) versus Con (n=98): in eyes closed (left) and open (right) conditions. The *P* values denote ranksum tests (not corrected for both comparisons).
B – Standard (above) and deviant (below) MMN responses for Con (n=94), PScz (n=96) and Rel (n=42) at electrode Fz. Red bars denote group differences in Con vs PScz, green bars denote group differences in Con vs Rel, both $P<0.05$ (*t*-tests at each timepoint, uncorrected).
C – Left: The lower plot shows the overall Con > PScz effect at sensor level, displayed at $P<0.05$ (FWE). The significant cluster highlights the group differences common to both standard and deviant conditions at around 100 ms (also visible in panel A). The location of electrode Fz is shown in black. The peak effect is shown in green: $P=0.003$ (FWE), $t(376)=4.83$. The upper plot is of anterior to posterior sensors vs time: the peak effect occurs at 82 ms.
Right: These plots show the overall Con > Rel effects at sensor level in the same format, displayed at $P<0.005$ (unc); the peak effect is at 94 ms, $P=0.001$ (unc), $t(268)=3.02$.
D – Group source localization of the MMN data, showing the three bilateral sources used in the DCM model (green crosses). NB this source localization is just for illustrative purposes: source localization was performed for each subject as part of the DCM inversion. Broca's area (left BA44 and BA45) is highlighted in the blue box.
E – These plots show the mean 40 Hz ASSR power spectra at electrode Fz for Con (n=92), PScz (n=94) and Rel (n=42). Each plot shows an example line of best fit (using robust fitting to the 10-30 Hz and 50-70 Hz ranges). These were used on an individual-subject basis to normalize their spectra by subtracting this 1/f gradient.
F – Unnormalized time frequency plots for the 40 Hz ASSR paradigm; the three rightmost plots show areas of group differences at the $P<0.05$ level (encircled) at each time point and frequency. Without normalization, there is no significant difference between Con and Rel at 40 Hz, but with normalization there is (Figure 4C).

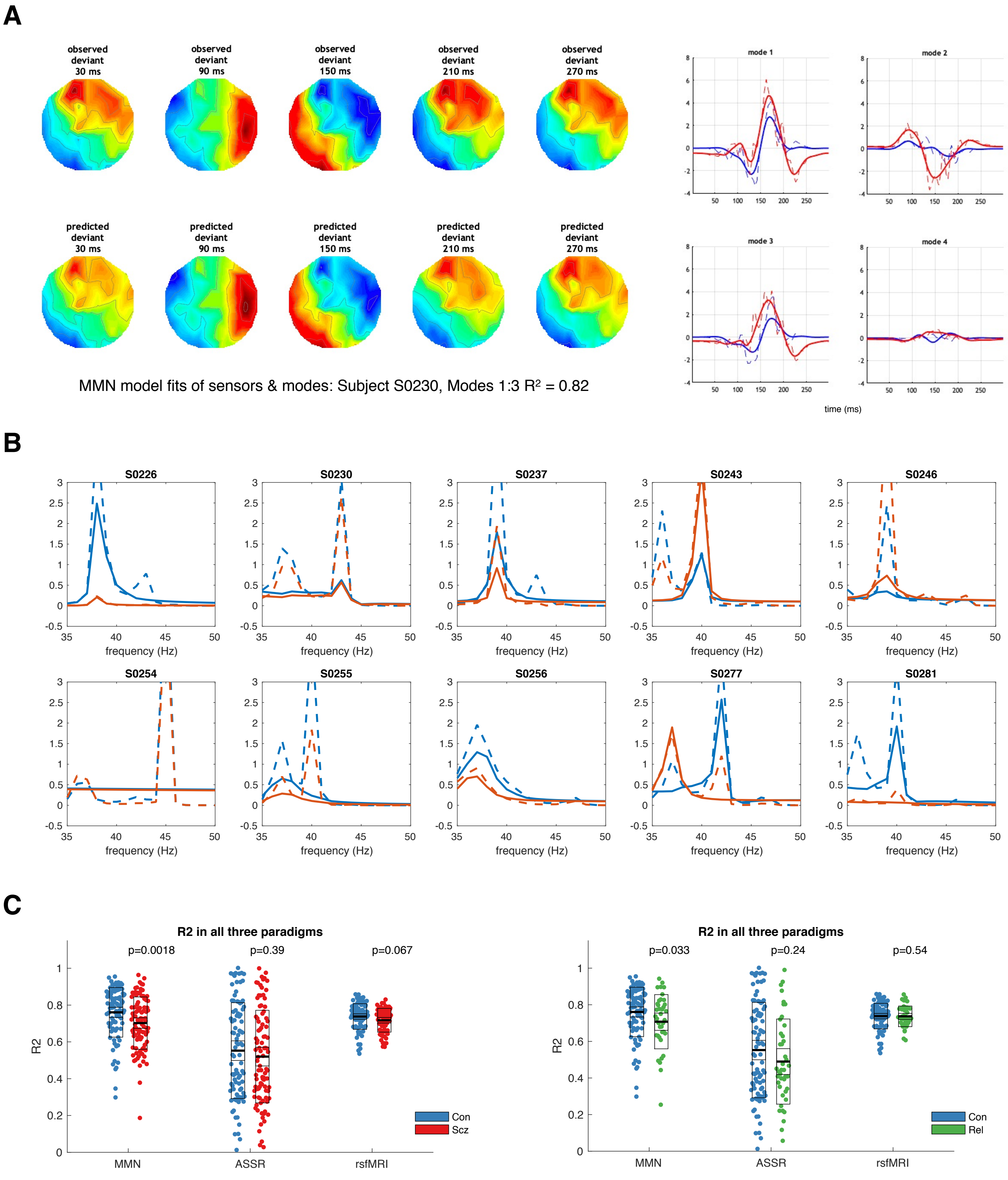

Figure S3 – MMN, 40 Hz ASSR and rsfMRI DCM model fits

A – Left: Example MMN model fits from a single subject (S0230) in sensor space. The top row shows the EEG data in the deviant condition at various timepoints; the bottom row shows the corresponding EEG data generated by the model parameters.
Right: Example MMN model fits of the first four (of eight) modes of the prior predicted covariance in sensor space. The standard and deviant conditions are plotted in blue and red respectively, data are plotted in dashed lines, the model fits in full lines. The first three modes capture most of the variance in the data.
B – 40 Hz ASSR model fits (full lines) for the first 10 subjects, for the power spectra (dashed lines) in left A1 (blue) and right A1 (orange). Fits are generally satisfactory – an exception is S0254, whose peak at >45 Hz is too far from the prior of 40 Hz for the model to accommodate.
C – These plots compare R^2 values for all three paradigms in Con and PScz (left) and Con and Rel (right) using ranksum tests as the distributions are skewed. *P* values are not corrected for multiple comparisons. In the MMN, Con have higher R^2 values than PScz (Z=3.16, P=0.0018), and Rel (Z=2.14, P=0.033). There are no significant differences in the other two paradigms.

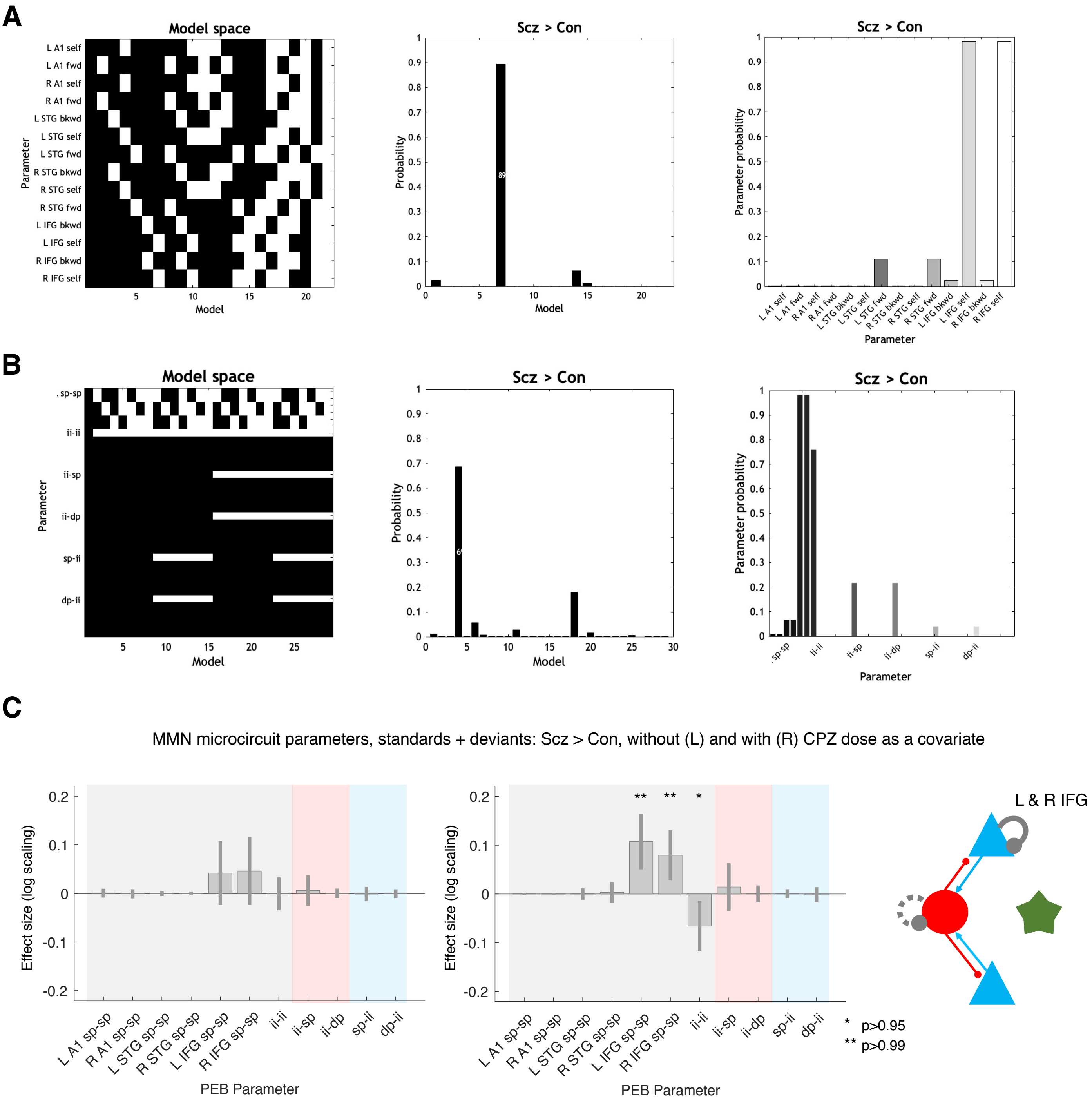

Figure S4 – MMN DCM model spaces and additional results

A – Left: The model space used to analyse the PScz > Con mismatch effect (and all other mismatch effects) within the PEB framework. The 22 rows each represent a model containing different combinations of extrinsic or self-inhibitory connection parameters (in white) that

could best explain the group difference in mismatch effects. The first model is a null model, i.e. that no parameters differ between groups.

Middle: The posterior probabilities of each of the 22 models are shown, following Bayesian model reduction. Model 7 (containing only bilateral IFG self-inhibitory connections) is the best model with a posterior probability of P=0.89.

Right: The posterior parameter probabilities are shown, following Bayesian model averaging. These are the probabilities that each parameter contributes to the group difference effect. The only two parameters of P>0.95 are self-inhibition in bilateral IFG.

B – These plots show the PEB analysis of the overall PScz > Con effect in the MMN (i.e. across both conditions) in the same format as above. There are 11 parameters in the model space (the same was used for all MMN microcircuit analyses): six sp-sp self-inhibitory parameters (one per region) and five more microcircuit parameters (identical in every region, hence they each appear only once in the rows). The results pertain to the analysis shown in Figure S4C (right).

C – These plots show overall PScz > Con differences in microcircuit parameters in the MMN (i.e. across both standard and deviant conditions), without (left) and with (right) the inclusion of chlorpromazine dose equivalent (CPZ) as a covariate of no interest, in the same format as Figure 3F. Without CPZ, the IFG effects only reach the P>0.75 level. With the inclusion of CPZ, however, PScz show increased sp self-inhibition (i.e. reduced synaptic gain) in bilateral IFG, and disinhibition of interneurons. CPZ is thus unlikely to be contributing to the increased sp self-inhibition seen in PScz: if anything, it might be ameliorating this deficit. These L and R IFG effects are also present without the addition of age, sex and smoking covariates (P>0.99).

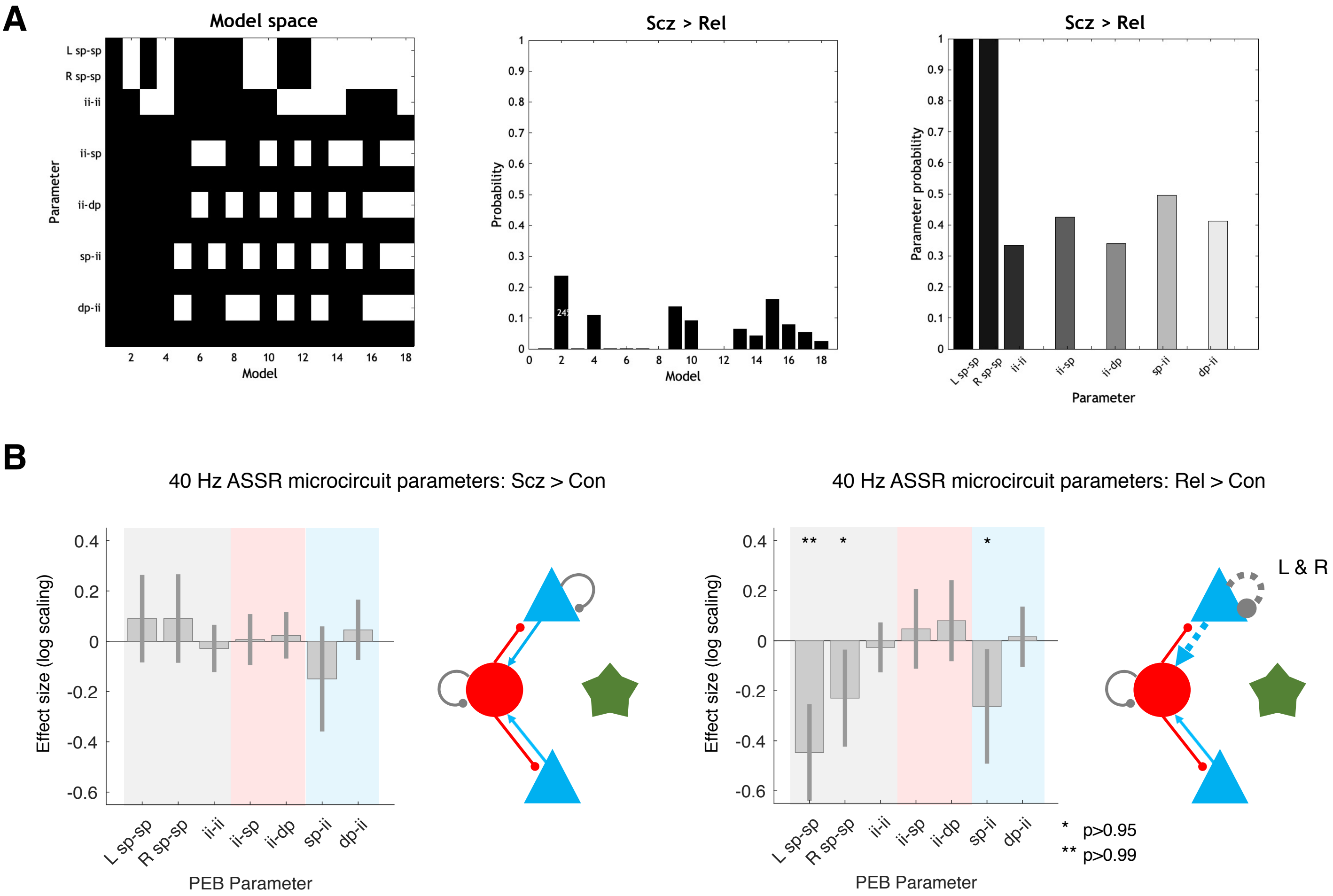

Figure S5 – 40 Hz ASSR DCM model space and additional results

A – These plots show the PEB analysis of the PScz > Rel effect in the 40 Hz ASSR in the same format as Figure S4A. The model space (identical in every 40 Hz ASSR analysis) contains different combinations of seven parameters: sp self-inhibition in L and R A1, and the five other microcircuit parameters (each constrained to be the same in both areas). No model is very likely (middle plot) but the L and R sp self-inhibition parameters appear in every one of the probable models, hence they are very likely differing between groups (right plot).

B – Left: The PScz > Con contrast did not show effects of *P*>0.95.

Right: Interestingly, the Rel > Con contrast indicated a loss of sp input into interneurons but also a disinhibition of sp cells – the former effect was also seen at *P*>0.75 in PScz, but the latter effect was reversed in PScz at *P*>0.75: see Figure 3C for an analysis combining all three groups. All effects shown are also present without the addition of age, sex and smoking covariates (*P*>0.95).

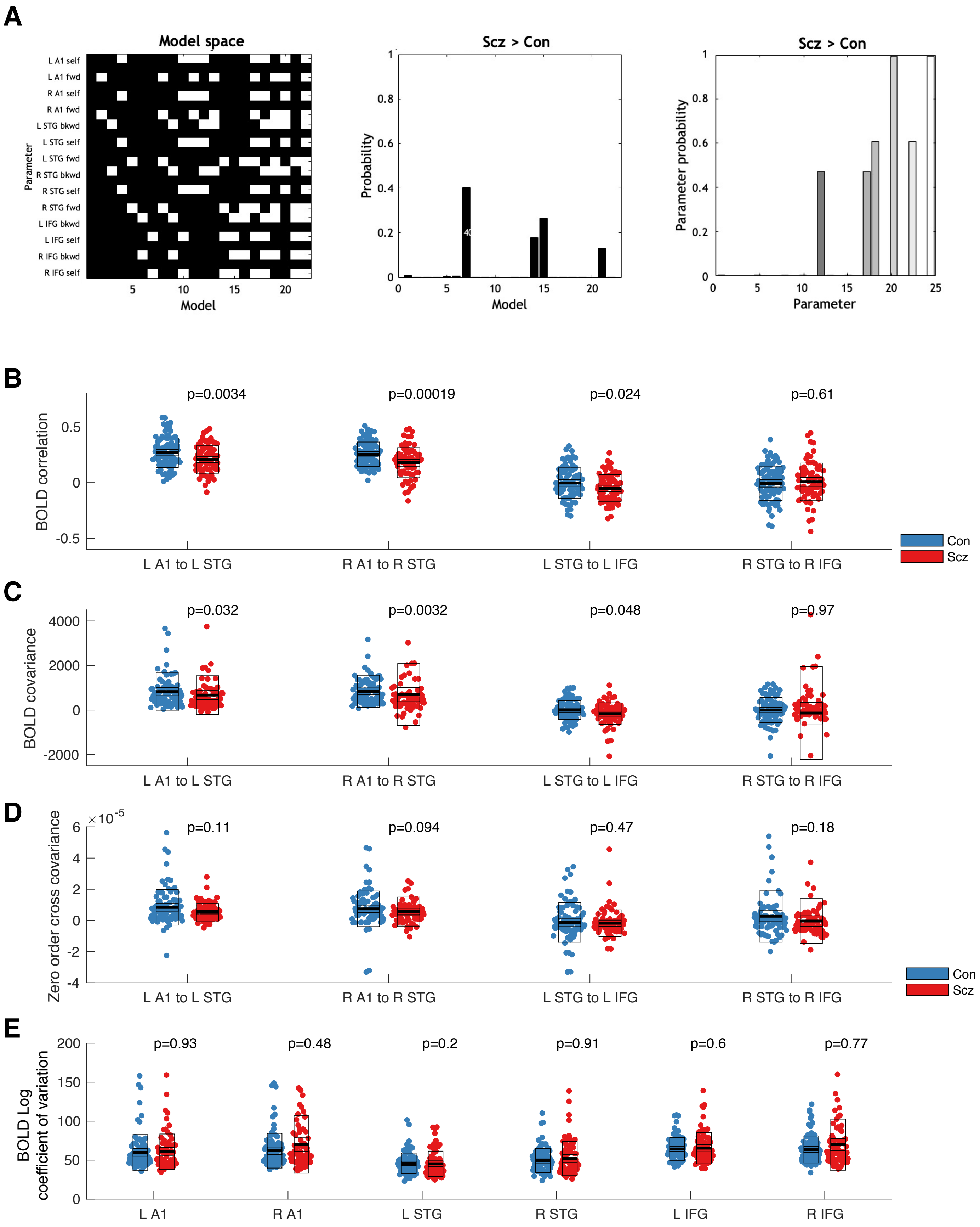

Figure S6 – resting state fMRI DCM model space and additional results

A – These plots show the PEB analysis of the PScz > Con effect in the rsfMRI paradigm in the same format as Figure S4A. The model space (identical in every rsfMRI analysis) contains different combinations of forward, backward and self-inhibitory connections in the same cortical network used in the MMN analysis.

B – Functional connectivity analysis (with global signal regression, GSR) between nodes of the MMN network in PScz (n=72) vs Con (n=85). There are significant decreases in functional connectivity (i.e. Pearson correlation) between A1 and STG bilaterally in PScz, and between L STG-IFG, although the latter would not survive multiple comparison correction (L A1-STG $t(154)=2.97$, $P=0.0034$; R A1-STG $t(154)=3.83$, $P=0.00019$; L STG-IFG $t(154)=2.29$, $P=0.024$, P values not Bonferroni-corrected). Note that these findings are quite different from the results of effective connectivity analysis in Figure 5A.

C – The functional connectivity analysis (with GSR) is repeated using covariance instead of correlation, and ranksum tests as the distributions are skewed. PScz vs Con differences are less marked (L A1-STG $Z=2.15$, $P=0.032$; R A1-STG $Z=2.95$, $P=0.0032$; L STG-IFG $Z=1.96$, $P=0.048$, P values not Bonferroni-corrected).

D – For comparison, the covariance (at zero lag, as in functional connectivity analysis) of the underlying neuronal activity inferred by DCM in each source is shown. There are no longer group differences (ranksum tests, all $P>0.05$).

E – (Log) coefficient of variation (standard deviation normalised by the mean) of the BOLD timeseries (with GSR) in each of the nodes of the network – no nodes show group differences (ranksum tests, all $P>0.1$), including L and R IFG, where self-inhibition is greater in PScz (Figure 5A).

See the Supplementary Methods for a discussion of how inferred effective connectivity may or may not be related to the corresponding functional connectivity between nodes (or variance within a node).

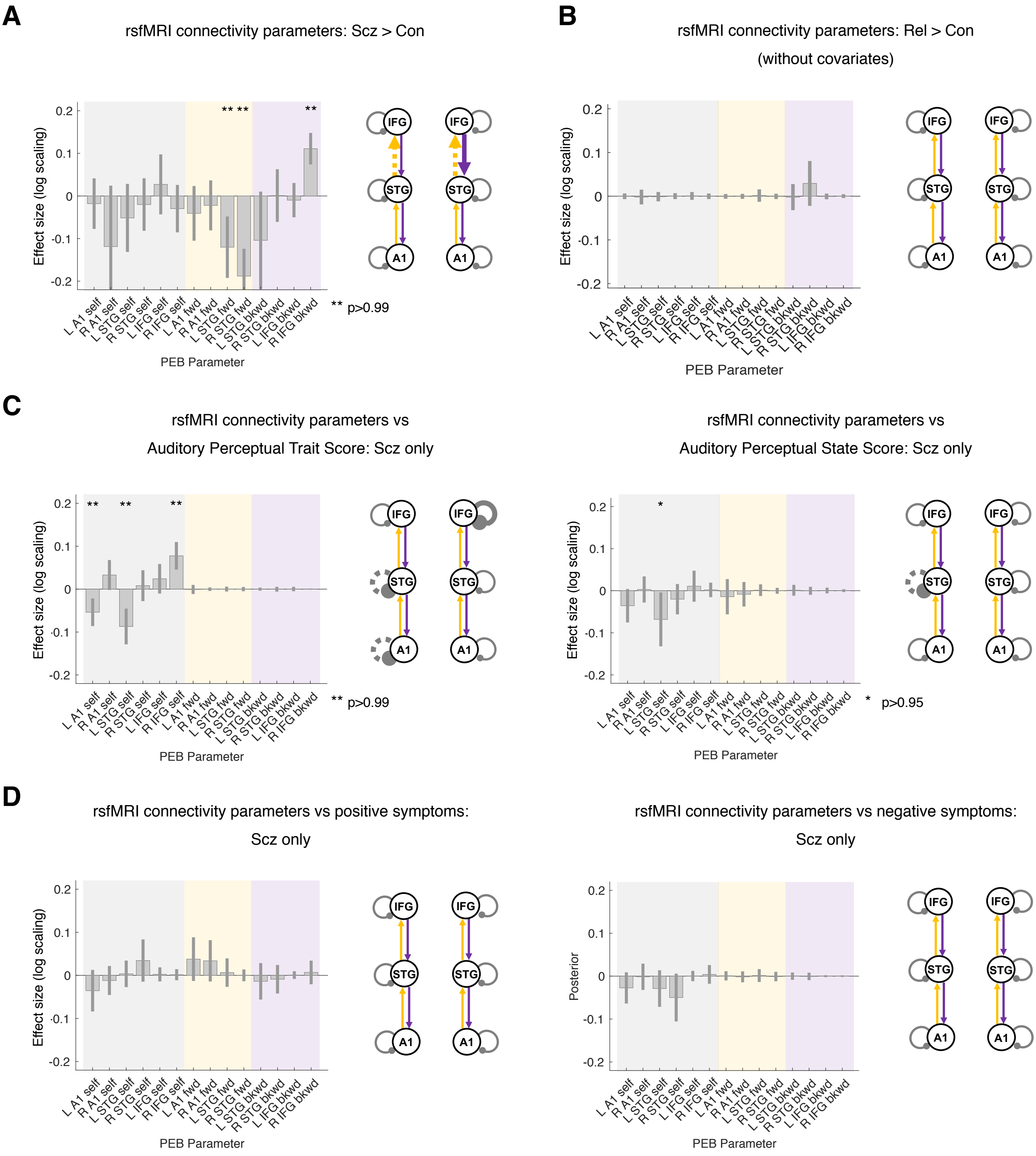

Figure S7 – Resting state fMRI results without global signal regression

These plots (in the same format as Figure 5) illustrate the importance of the global signal regression (GSR) preprocessing step in the rsfMRI analysis of PScz (n=72) and Con (n=85): many results change substantially without it.

A – With GSR, PScz show a loss of gain in bilateral IFG, but without GSR, PScz show a loss of forward frontotemporal connectivity instead.
B – With GSR, Rel show a loss of gain in bilateral IFG (without inclusion of the age covariate), but without GSR, no effects are significant (without the age covariate).
C – Left: With GSR, abnormal auditory percepts 'trait' score in PScz relate to loss of gain in bilateral IFG; without GSR, this effect is still seen in R IFG, but there are also effects of disinhibition in L temporal cortex (similar to state abnormal auditory perceptions both with and without GSR).
Right: With GSR, abnormal auditory percepts 'state' score in PScz relate to disinhibition in bilateral temporal lobes and loss of STG-A1 connectivity also; without GSR, only the disinhibition in L STG survives.
D – Left: With GSR, BPRS positive symptoms related to disinhibition in five regions and increased forward connectivity in three connections; without GSR, only disinhibition in L A1 and increased forward connectivity from bilateral A1 to STG are seen, at $P>0.75$. Right: With GSR, BPRS negative symptoms related to disinhibition in all temporal nodes; without GSR, no effects survive.

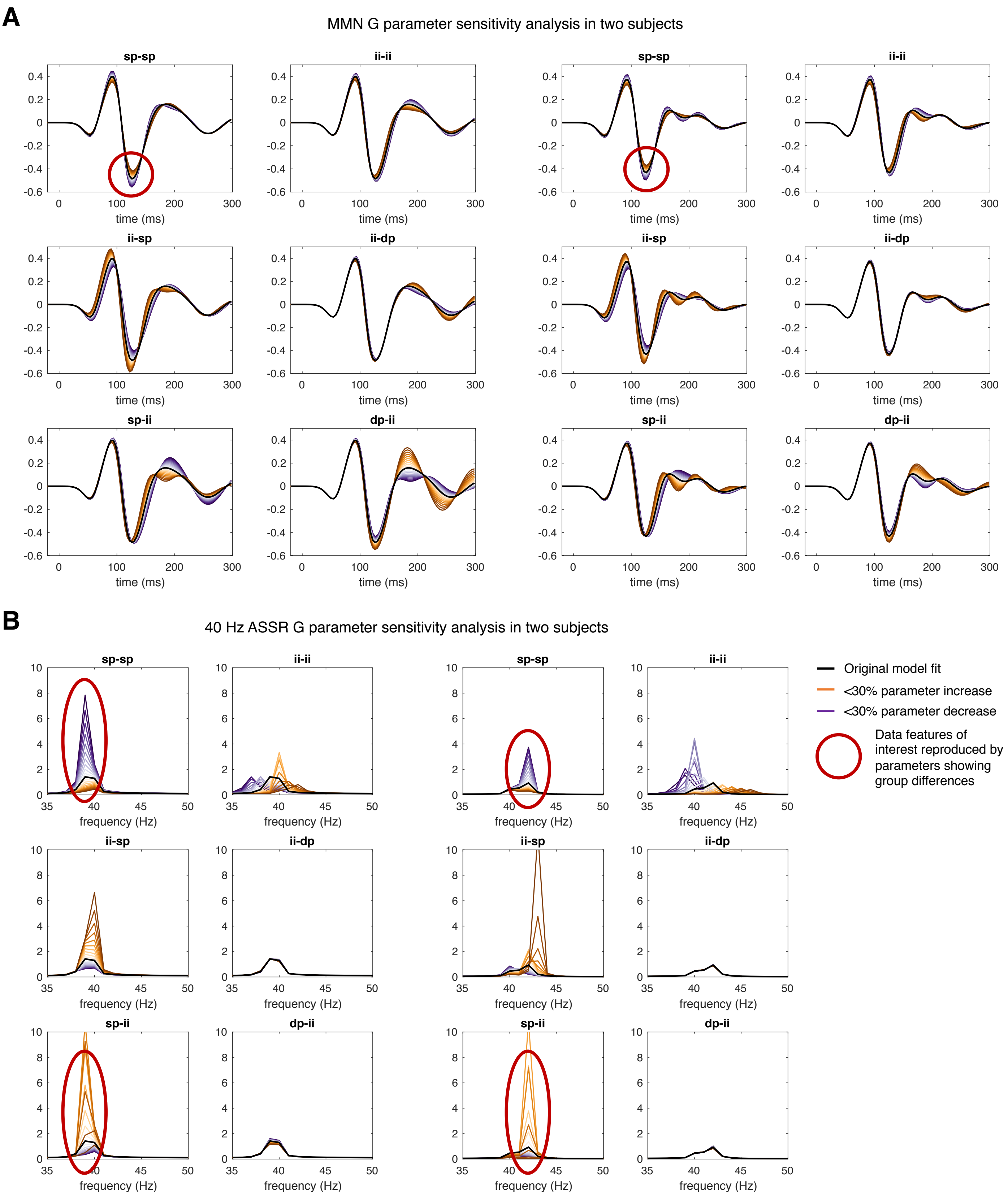

Figure S8 – EEG microcircuit parameter sensitivity analyses

This figure shows the results of simulating virtual electrode data in a single cortical area to reproduce key data features in the MMN and 40 Hz ASSR paradigms. In each paradigm, two subjects representative of the group average were selected, and their posterior G (intrinsic connectivity) parameters used to simulate data. In each simulation, one G parameter in turn was progressively changed by ±30% – results of increases and decreases are shown in orange and purple, with darker shades representing bigger changes. The left and right panels correspond to the two subjects in each paradigm, and the six plots in each panel to the six G parameters: sp-sp, ii-ii, ii-sp, ii-dp, sp-ii and dp-ii connectivities. Note that the first two are self-inhibitory connections (synaptic gain).

A – Sensitivity analysis in the MMN paradigm. The ERP data showed a reduced MMN amplitude (Figure 3A) and P100 amplitude (Figure S2A) but no significant change in MMN latency in PScz; in PScz, sp-sp was increased (Figure 3F). In both subjects' simulations, increasing sp-sp (i.e. reducing synaptic gain) reduces the amplitude of the negative deflection (circled in red) without changing the latency, as it also does to the P100. Increasing ii-ii has similar but smaller effects on these amplitudes, but shortens the latency. Decreasing ii-sp likewise reduces the amplitudes, but lengthens the latency. The remaining parameters cause more substantial changes in the latter half of the waveform. The waveform itself is compressed in time compared with empirical data, because extrinsic connections – which cause a ~10 ms delay per connection – are not included in the simulation of a single area.

B – Sensitivity analysis in the 40 Hz ASSR paradigm. The power spectral data showed a reduction in ~40 Hz power (Figure 4C). In both subjects' simulations, increasing sp-sp and decreasing sp-ii (the parameter changes in PScz vs Rel and PScz+Rel vs Con respectively – Figure 4G) both reduce ~40 Hz power (circled in red). Reducing ii-sp has similar but less powerful effects on sp-ii. Changing ii-ii changes the peak frequency, but its effects on power are only prominent at precisely 40 Hz. Dp-ii and ii-dp connections have minimal effects.

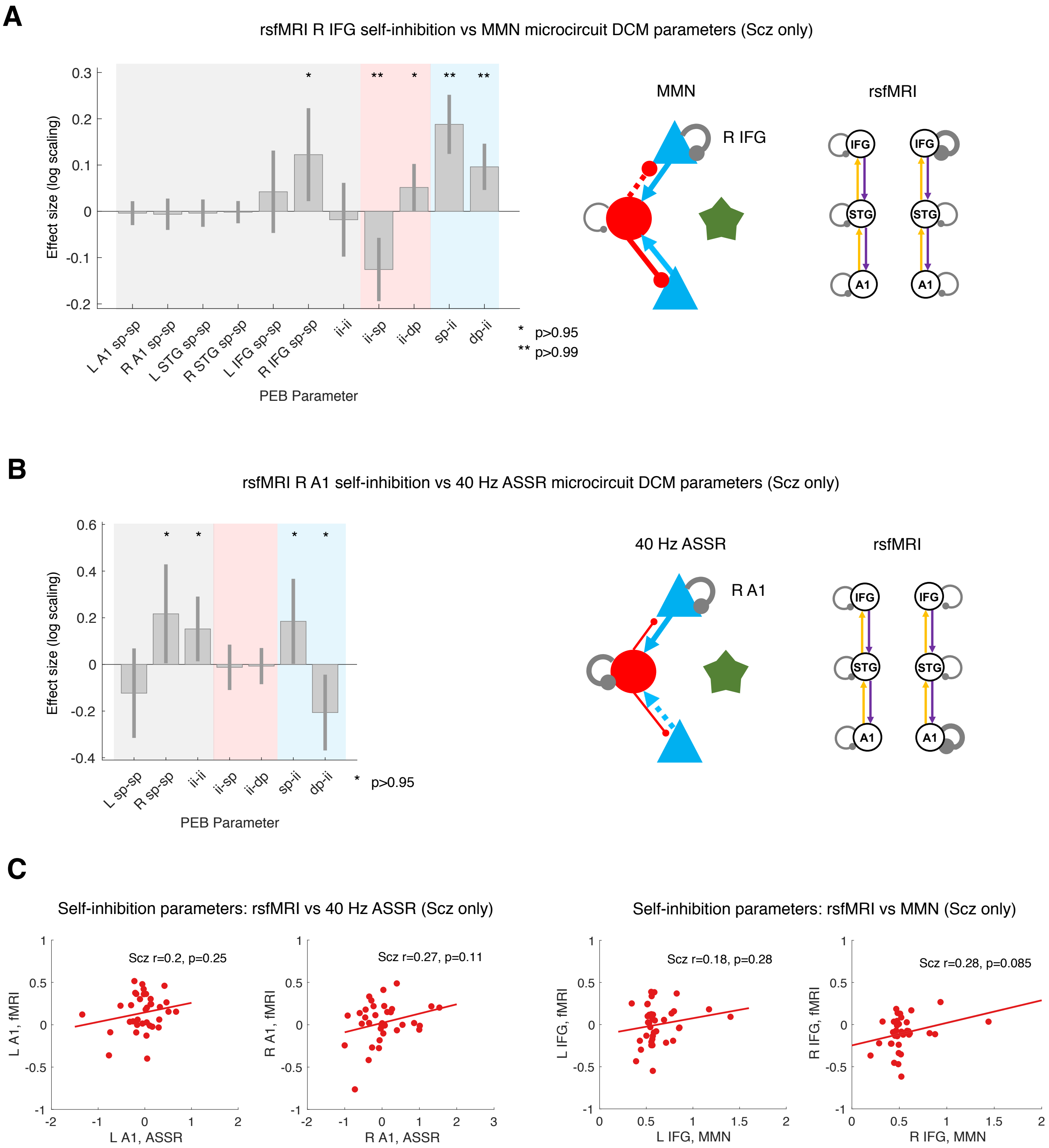

Figure S9 – Resting state fMRI & EEG DCM parameter relationships in PScz

All analyses are conducted on PScz whose rsfMRI and EEG paradigms were recorded <100 days apart (for MMN, n=44; for 40 Hz ASSR, n=40).

A – In PScz, increased self-inhibition in R IFG (estimated from rsfMRI) relates to both loss of superficial pyramidal cell gain in R IFG specifically and a net increase of inhibition of pyramidal cells (i.e. stronger pyramidal inputs to interneurons and stronger returning connections except for ii-sp, whose decrease is smaller than the sp-ii increase) in the MMN analysis. All effects (except the ii-dp increase) shown are also present without the addition of age, sex and smoking covariates (P>0.95).
B – In PScz, increased self-inhibition in R A1 (estimated from rsfMRI) relates to increased sp self-inhibition in R A1 specifically and stronger sp input to interneurons. It also – counterintuitively – relates to greater self-inhibition of and weaker dp input to interneurons. All effects (except the dp-ii decrease) shown are also present without the addition of age, sex and smoking covariates (P>0.95).
NB With the inclusion of chlorpromazine dose equivalents as a covariate the specific R IFG effect (in A) and R A1 effect (in B) are lost, however, and likewise if PScz with rsfMRI and EEG paradigms >100 days apart are included.
C – These plots show the correlations between self-inhibition parameters across EEG and rsfMRI paradigms within PScz. The left two plots show correlations between 40 Hz ASSR and rsfMRI estimates of self-inhibition in bilateral A1, the right two plots show correlations between MMN and rsfMRI estimates of self-inhibition in bilateral IFG. Relationships in R A1 and R IFG approached statistical significance, and are analysed in more detail using PEB in panels A and B above. There were no correlations between the variance of these parameters across these combinations of paradigms (all P>0.3).

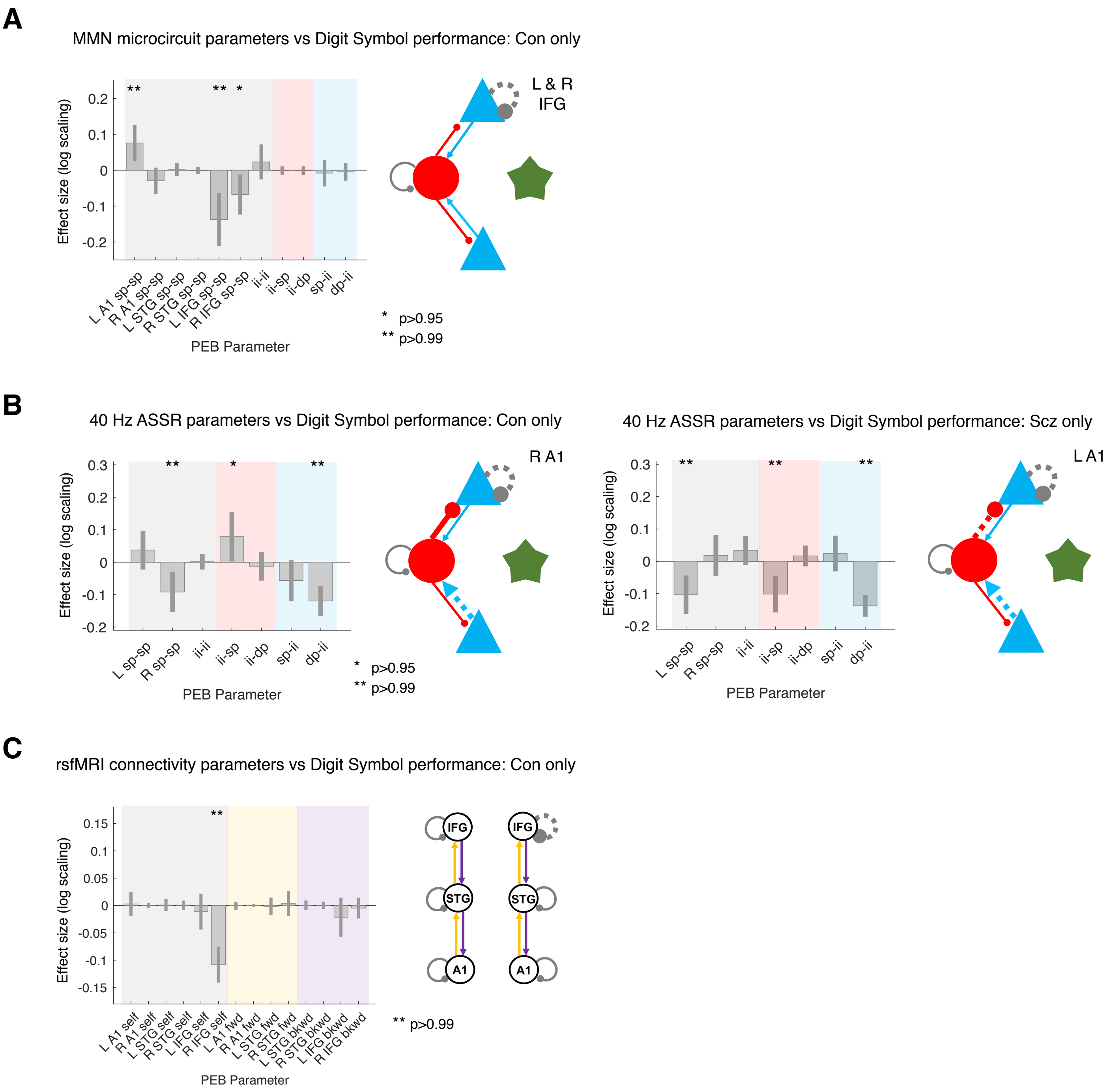

Figure S10 – Relationships of DCM self-inhibition parameters to Digit Symbol task performance

A – A PEB analysis of MMN microcircuit model parameters that correlate with Digit Symbol score within Con only. The results plotted on the left correspond to microcircuit parameters (illustrated on the right): blue shaded connections are superficial and deep pyramidal inputs to interneurons, red shaded connections are interneuron outputs to pyramidal layers, and grey

shaded connections are self-inhibitory. Self-inhibitory connections are estimated in interneuron and superficial pyramidal populations: the latter differ in every cortical area (all other microcircuit parameters are identical in all cortical areas). Better performance on the Digit Symbol task relates to reduced self-inhibition (i.e. increased synaptic gain) of superficial pyramidal cells in IFG (indicated using dotted lines on the schematic; the result in A1 is not shown).

B – PEB analyses in Con (left) and PScz (right) groups, showing Digit Symbol score relates to reduced sp self-inhibition (i.e. increased synaptic gain), and lower dp-ii connectivity.

C – PEB analysis of the rsfMRI showed that in Con, Digit Symbol score related to reduced self-inhibition in R IFG. PScz results are not shown.

The relationship between Digit Symbol (i.e. processing speed) performance and reduced self-inhibition is remarkably consistent across paradigms in Con, but not in PScz. All effects shown are also present without the addition of age, sex and smoking covariates (P>0.95), and also with inclusion of chlorpromazine dose equivalents as a covariate.

Table S1: Participant characteristics

	Controls	PScz	Relatives	Con v PScz	Con v Rel	Rel v PScz
Total	107	108	57			
**Demographics**						
Age, years (mean ±std)	39.4 ±14.3	39.4 ±13.9	45.4 ±16.6	p=1, t=0.0	**p=0.02, t=2.4**	**p=0.01, t=2.5**
Sex	64 M, 43 F	73 M, 35 F	16 M, 41 F	p=0.2, χ=1.4	**p=10^{-4}, χ=15**	**p=10^{-6}, χ=23**
Smoking (current)	35 (33%)	42 (39%)	9 (16%)	p=0.3, χ=0.9	**p=0.02, χ=5.4**	**p=10^{-3}, χ=9.3**
**Psychological measures**						
Digit Symbol (mean ±std)	10.5 ±3.1	7.6 ±3.0	10.2 ±3.1	**p=10^{-9}, t=6.5**	p=0.6, t=0.5	**p=10^{-6}, t=4.9**
APTS Trait (mean ±std) (min 0, max 48)	4.4 ±5.4	18.2 ±13.4	4.2 ±4.6	**p=0, t=9.8**	p=0.8, t=0.2	**p=10^{-11}, t=7.5**
APTS State (mean ±std) (min 0, max 48)	0.9 ±2.0	10.0 ±11.7	1.5 ±3.3	**p=10^{-13}, t=7.9**	p=0.2, t=1.2	**p=10^{-7}, t=5.3**
**Clinical measures**						
Antipsychotic prescribed	0	90	1			
Antipsychotic types	67 atypical*, 15 typical*, 22 Clozapine*, 3 unmedicated, 15 not recorded<br>*includes 15 on combinations of these					
CPZ equivalent, mg (mean ±std)	n/a	464 ±488	3.5 ±26.3			
Antidepressant prescribed	13	39	9			
Benzodiazepine (not daily) prescribed	5	16	3			
Mood stabiliser prescribed	3	21	1			
BPRS total (mean ±std) (scale from 20 to 140)	n/a	38.8 ±12.3	n/a			
BPRS positive* (mean ±std) (scale from 7 to 49)	n/a	14.4 ±7.4	n/a			
BPRS negative** (mean ±std) (scale from 4 to 28)	n/a	7.3 ±4.5	n/a			
**Imaging paradigms**						
rsEEG analysed/obtained	98/107	95/108	n/a	p=0.8, χ=0.4		
MMN modelled/obtained	93/98	95/100	40/48	p=1, χ=0.0	**p=0.02, χ=5.3**	**p=0.02, χ=5.3**
ASSR modelled/obtained	92/101	94/105	42/53	p=0.7, χ=0.1	**p=0.04, χ=4.3**	p=0.08, χ=3.1
rsfMRI modelled/obtained	85/107	72/108	45/54	**p=0.03, χ=4.5**	p=0.6, χ=0.4	**p=0.03, χ=5.0**
rsfMRI motion scrubbed frames, % (mean ±std)	4.1 ±6.2	6.4 ±9.9	5.0 ±8.6	p=0.08, t=1.7	p=0.5, t=0.7	p=0.5, t=0.7
rsfMRI SNR	46.6 ±9.2	43.5 ±13.0	43.9 ±10.7	p=0.09, t=1.7	p=0.1, t=1.5	p=0.9, t=0.2
Modelled rsfMRI & MMN within 100 days/max days	51/75	37/64	22/33			
Days between rsfMRI & MMN in modelled subjects scanned <100 days apart (mean, interquartile range)	35 days [11 51]	26 days [4 49]	31 days [6 47]			
Modelled rsfMRI & ASSR within 100 days/max days	52/73	41/61	26/35			
Days between rsfMRI & ASSR in modelled subjects scanned <100 days apart (mean, interquartile range)	22 days [8 29]	18 days [4 23]	24 days [8 39]			

*BPRS positive score = sum of items 4, 7, 8, 11, 12, 15, 20 on the 20 item BPRS. Mean ±std in PScz were
4 – Conceptual disorganization: 2.1 ±1.5; 7 – Mannerisms and posturing: 1.6 ±1.1
8 – Grandiosity: 1.6 ±1.2; 11 – Suspiciousness: 2.8 ±1.9
12 – Hallucinatory behaviour: 2.9 ±1.8; 15 – Unusual thought content: 3.0 ±1.7
20 – Inappropriate affect: 1.4 ±0.9

**BPRS negative score = sum of items 3, 13, 16, 19 on the 20 item BPRS
